# Fragment Screening Reveals Starting Points for Rational Design of Galactokinase 1 Inhibitors to Treat Classic Galactosemia

**DOI:** 10.1021/acschembio.0c00498

**Published:** 2021-03-16

**Authors:** Sabrina R. Mackinnon, Tobias Krojer, William R. Foster, Laura Diaz-Saez, Manshu Tang, Kilian V. M. Huber, Frank von Delft, Kent Lai, Paul E. Brennan, Gustavo Arruda Bezerra, Wyatt W. Yue

**Affiliations:** † Structural Genomics Consortium, Nuffield Department of Medicine, University of Oxford, Oxford, United Kingdom, OX3 7DQ; ‡ Department of Pediatrics, University of Utah, Salt Lake City, Utah 84108-6500, United States; § Diamond Light Source, Harwell Science and Innovation Campus, Didcot, Oxfordshire, United Kingdom, OX11 0DE; ∥ Target Discovery Institute, University of Oxford, Oxford, United Kingdom, OX3 7FZ

## Abstract

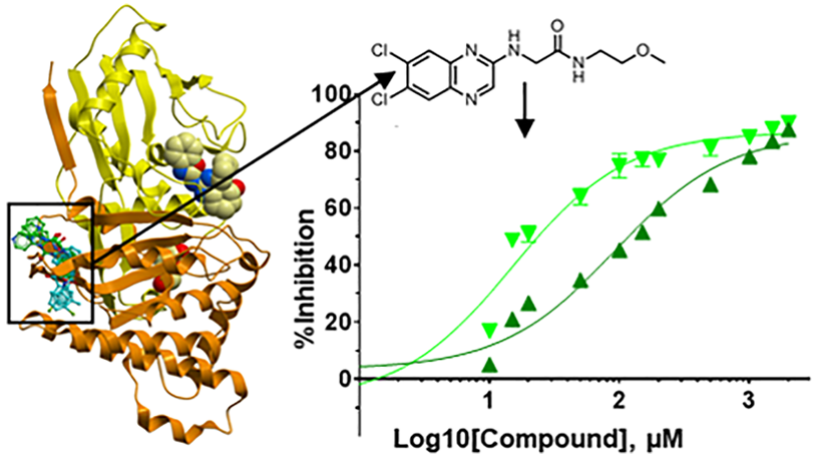

Classic galactosemia is caused by loss-of-function mutations in galactose-1-phosphate uridylyltransferase (GALT) that lead to toxic accumulation of its substrate, galactose-1-phosphate. One proposed therapy is to inhibit the biosynthesis of galactose-1-phosphate, catalyzed by galactokinase 1 (GALK1). Existing inhibitors of human GALK1 (hGALK1) are primarily ATP-competitive with limited clinical utility to date. Here, we determined crystal structures of hGALK1 bound with reported ATP-competitive inhibitors of the spiro-benzoxazole series, to reveal their binding mode in the active site. Spurred by the need for additional chemotypes of hGALK1 inhibitors, desirably targeting a nonorthosteric site, we also performed crystallography-based screening by soaking hundreds of hGALK1 crystals, already containing active site ligands, with fragments from a custom library. Two fragments were found to bind close to the ATP binding site, and a further eight were found in a hotspot distal from the active site, highlighting the strength of this method in identifying previously uncharacterized allosteric sites. To generate inhibitors of improved potency and selectivity targeting the newly identified binding hotspot, new compounds were designed by merging overlapping fragments. This yielded two micromolar inhibitors of hGALK1 that were not competitive with respect to either substrate (ATP or galactose) and demonstrated good selectivity over hGALK1 homologues, galactokinase 2 and mevalonate kinase. Our findings are therefore the first to demonstrate inhibition of hGALK1 from an allosteric site, with potential for further development of potent and selective inhibitors to provide novel therapeutics for classic galactosemia.

## Introduction

The Leloir pathway is essential for the metabolism of dietary galactose,^1^ generating glucose units for glycolysis and biosynthesis of glycogen, glycoproteins, and glycolipids. At the hub of the Leloir pathway is galactose-1-phosphate uridylyltransferase (GALT; EC 2.7.7.12), which converts galactose-1-phosphate (Gal-1-P) and UDP-glucose (UDP-Glc) into glucose-1-phosphate and UDP-galactose.^2^ Inherited mutations of the *GALT* gene lead to the autosomal recessive disorder classic galactosemia (OMIM 230400),^3^ affecting 1:16 000–60 000 live births. Classic galactosemia patients generally sicken in the neonatal period, with liver, kidney, intestinal, and central nervous system toxicity exacerbated by the high galactose content in human and formula milk.^4,5^ If lactose, the primary exogenous source of galactose, is not removed from the patient’s diet, progressive liver and brain damage lead to death or severe disability. The current mainstay treatment is life-long dietary galactose restriction, which resolves acute life-threatening symptoms but fails to prevent the long-term, late-onset complications that include learning and speech difficulties, neurological impairments manifesting as ataxia, and premature ovarian insufficiency.^4^

In classic galactosemia, GALT enzymatic deficiency causes the accumulation of Gal-1-P, the product of the enzyme galactokinase 1 (GALK1; EC 2.7.1.6) upstream of GALT in the Leloir pathway. Gal-1-P is proposed to be the major, if not sole, pathogenic driver of disease.^6^ Gal-1-P has been shown to inhibit various metabolic enzymes with key cellular functions, including inositol phosphatase,^7^ UDP-Glc pyrophosphorylase and galactosyltransferases,^8−11^ glycogen phosphorylase, and phosphoglucomutase.^12,13^ Inhibition of human GALK1 (hGALK1) to prevent Gal-1-P production therefore could provide a therapeutic benefit for GALT deficiency.^14^ This is supported by the evidence that *galk1* knockout in Drosophila rescued the galactosemic neurological phenotype,^15^*galk1* knockdown in GALT-deficient yeast abolished sensitivity to galactose levels,^16,17^ and patients with inherited GALK1 deficiency (OMIM 230200) present milder phenotypes (e.g., early onset cataracts) and do not accumulate Gal-1-P.^18^ This “substrate reduction” approach, to mitigate the toxic accumulation of a metabolite that arises from a metabolic block by inhibiting an enzyme upstream of it, is gaining therapeutic potential for other inborn errors of metabolism.^19,20^

**Figure 1:**
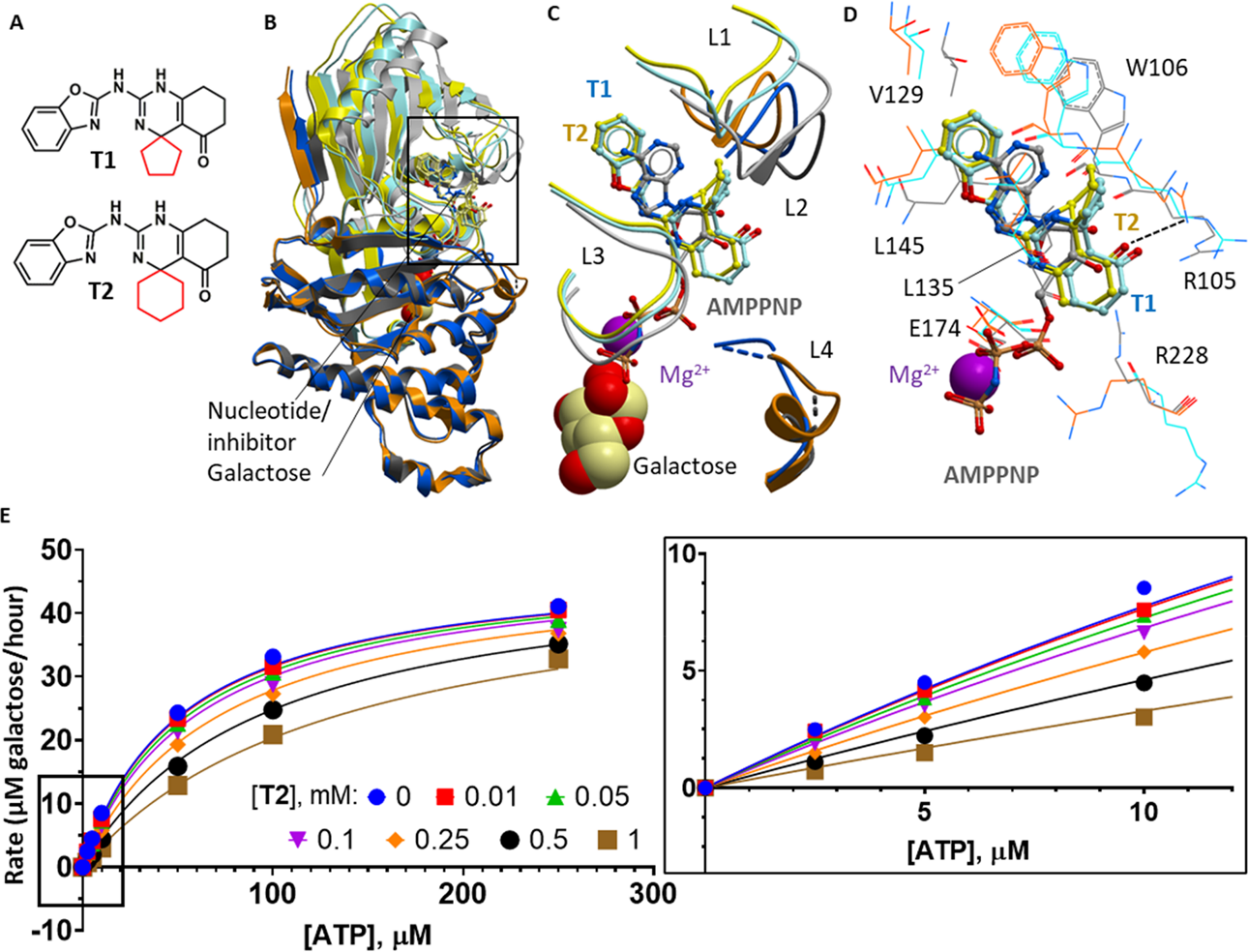
Structural and kinetic characterization of spiro-benzoxazole inhibitors of GALK1. (A) Chemical structures of the two spiro-benzoxazole inhibitors characterized in this work. The spiro-ring, differing between the two compounds, is highlighted in red. (B) Superimposed structures of GALK1-galactose-AMPPNP (PDB 1WUU; light/dark gray), GALK1-galactose-**T1** (light/dark blue), and GALK1-galactose-**T2** (yellow/orange) structures. The N-terminal domain is shaded lighter than the C-terminal domain. Galactose and magnesium are shown as spacefill; AMPPNP, **T1**, and **T2** are shown as gray, blue, and yellow sticks, respectively. (C) Close-up view of the boxed area shown in panel B. The four ATP binding loops (L1, Ser79–Pro85; L2, Ser98–Arg105; L3, Val133–Ser140; and L4; Arg228–Glu235) are shown as ribbons. Galactose and magnesium ions are shown as spacefill, and AMPPNP, **T1**, and **T2** are shown as gray, blue, and yellow sticks, respectively. (D) Close-up view of the GALK1 active site illustrating the binding mode of AMPPNP, **T1**, and **T2**. Nearby protein residues of the GALK1–galactose–AMPPNP, GALK1–galactose–**T1**, and GALK1–galactose–**T2** structures are shown as gray, blue, and orange lines, respectively. (E) Least-squares nonlinear fit of GALK1 reaction rate (total galactose consumed after 1 h reaction, μM) against increasing ATP concentrations (0–250 μM) in the presence of different concentrations of the inhibitor **T2** (0–1 mM). Curves were fitted to the competitive inhibition model, the best fitting enzyme kinetics–inhibition equation, using the GraphPad Prism software. Inset: Close-up view of plot showing GALK1 reaction rate (total galactose consumed after 1 h reaction, μM) against increasing ATP concentrations (0–10 μM) in the presence of different concentrations of the inhibitor **T2** (0–1 mM).

GALK1 is a small molecule kinase of the GHMP kinase family,^21^ which catalyzes the MgATP-dependent phosphorylation of the C-1 hydroxyl of α-d-galactose to yield Gal-1-P in the Leloir pathway. The crystal structure of hGALK1 in complex with galactose and the nonhydrolyzable ATP analogue AMPPNP (PDB 1WUU) supported an ordered kinetic mechanism, whereby ATP stabilizes the active site loops (L1–L4, shown in Figure 1) to facilitate galactose binding.^22,23^ Current inhibitors in development for hGALK1 are ATP-competitive. For example, a quantitative high-throughput screening (qHTS) campaign using a luminescence-based activity assay yielded a series of spiro-benzoxazole derivatives, such as **T1** and **T2** (Figure 1A), which are analogues of ATP, achieving potency in the low-to-mid micromolar range following compound optimization.^24,25^ No inhibitor-bound co-crystal structures of hGALK1 have been reported prior to this work.

This study presents the first structural evidence that the spiro-benzoxazole inhibitors **T1** and **T2** target the ATP pocket of hGALK1. To discover novel chemical starting points for hGALK1 inhibitors, we embarked on crystallography-based fragment screening,^26,27^ which has not been reported previously for galactosemia drug discovery. To bias hits toward allosteric binding, we soaked compounds into hGALK1 co-crystals with galactose and **T2** occupying the active site. We identified hits bound to a previously uncharacterized nonorthosteric pocket of hGALK1, resulting in hGALK1 inhibition at micromolar potency. These molecules inhibit hGALK1 activity in a manner that is not competitive toward its substrates and is selective over other members of the GHMP kinase family.

## Results and Discussion

### Co-crystal Structures with ATP-Competitive hGALK1 Inhibitors

A series of spiro-benzoxazole inhibitors of low-micromolar potency was previously reported for hGALK1, and *in silico* modeling and kinetic characterization were indicative of their binding in the active site ATP pocket,^24,25,28^ although no experimental structures have been reported. To this end, we co-crystallized hGALK1 with two spiro-benzoxazole inhibitors (**T1** and **T2**, Figure 1A), in the presence of galactose, and determined their crystal structures to 2.44 and 2.10 Å resolution, respectively (Table S1).

**T1** and **T2** share the chemical scaffold of a benzoxazole ring connected to a tetrahydroquinazolinone ring by an amide linker, and both compounds are found to occupy the ATP binding site in a similar manner (Figure 1B,C). The benzoxazole ring, mimicking the adenine moiety of ATP, sits in a hydrophobic pocket forming a π–π stacking interaction with Trp106 and van der Waals interactions with Val129 and Leu145 (Figure 1D). The amide linker and dihydropyrimidine substituent of the quinazolinone ring occupy a similar position to that of the ATP ribose moiety. Both compounds elicit changes in the four active site loops involved in ATP binding (Figure 1C). Importantly, the quinazolinone cyclohexanone and spiro-ring substituents cause displacement of two key side-chains—Arg105 and Arg228—at the entrance of the ATP site.

Molecular dynamics simulations have implicated Arg105 and Arg228 in providing the oxyanion hole to facilitate phosphoryl transfer from ATP to galactose.^28,29^ Under this mechanism, Arg105 would directly interact with ATP phosphate groups, while Arg228 would stabilize the negative charge developed at the β,γ-bridging oxygen of the ATP during bond cleavage. In our costructures, both Arg105 and Arg228 are sequestered away from the ATP phosphate groups, to accommodate the quinazolinone ring of the compounds (Figure 1D). For **T2** with the larger spiro ring, the carbonyl moiety is close enough to hydrogen bond with the Arg105 guanidino side chain (dotted line, Figure 1D). Therefore, these residues are sequestered away from catalysis in the inhibitor-bound complexes. Because Arg228 is not conserved among GHMP kinases, exploiting an interaction with this residue could confer selectivity for the development of hGALK1-specific inhibitors.^29^

We reproduced the inhibition of hGALK1 by the spiro-benzoxazole inhibitors using the Kinase-Glo luminescence-based assay (Figure S2), previously applied in HTS campaigns for hGALK1.^14,24,25,30^ In this method, the level of ATP remaining after the hGALK1 reaction is measured as a function of luciferin turnover and subsequent light generation caused by the ATP-dependent activity of luciferase enzyme.^31^ We observed dose-dependent inhibition of hGALK1, with IC_50_’s (**T1**: IC_50_ 12 μM, pIC50 4.9 ± 0.2; **T2**: IC_50_ 17 μM, pIC50 4.8 ± 0.1) similar to published values^24^ (Figure S2). This assay setup is not suited for measuring hGALK1 activity at higher ATP concentrations, however, as the luciferase enzyme is subjected to feedback inhibition by its product oxyluciferin.^32^

Consequently, to confirm the mode of inhibition of the spiro-benzoxazole inhibitors with respect to ATP, we adopted the Amplex Red fluorescence-based assay^33,34^ by coupling the hGALK1 reaction with *D. dendroides* galactose oxidase (GAO; EC 1.1.3.9) to measure the level of galactose remaining after being depleted as a substrate for hGALK1. In this method, hydrogen peroxide generated by the oxidation of galactose by GAO is used by horseradish peroxidase to convert the nonfluorescent substrate Amplex Red into its highly fluorescent product resorufin; therefore, the resultant fluorescent signal is inversely proportional to hGALK1 enzyme activity. We optimized several parameters of this assay to allow measurement of hGALK1 activity for the first time (Figure S3; Supplementary Results). ATP-titration experiments with **T2** showed no significant change in Vmax_app_ (*P* = 0.1801) and a significant increase in *K*m_app_ (*P* < 0.0001), collectively indicating competitive inhibition with ATP by **T2**, which is supported by fitting in GraphPad Prism (Figure 1E) and is in agreement with its binding mode in our hGALK1 crystal costructure.

### Developing a Robust Crystal System for hGALK1 Fragment Screening

As hGALK1 belongs to the kinase superfamily, we recognize that developing inhibitors that are not ATP-competitive could be beneficial for downstream optimization of pharmacokinetics/dynamics. To this end, we pursued the fragment screening approach, motivated by its emerging promise in revealing novel binding pockets and providing good chemistry starting points.^35^ We coupled this approach with X-ray crystallography, to provide direct structural readout as part of the screening process.^36^ We found that hGALK1 co-crystallized with galactose and **T2** produced the required quantity (crystals in the hundreds) and diffraction quality (consistently better than 2.5 Å) for the screening campaign. Additionally, this crystal system is biased for the detection of fragments binding to nonorthosteric sites of hGALK1 outside of the ATP and galactose pockets. hGALK1 crystals were each soaked with an individual fragment from the DSi-Poised library^26^ at a millimolar concentration.

**Figure 2:**
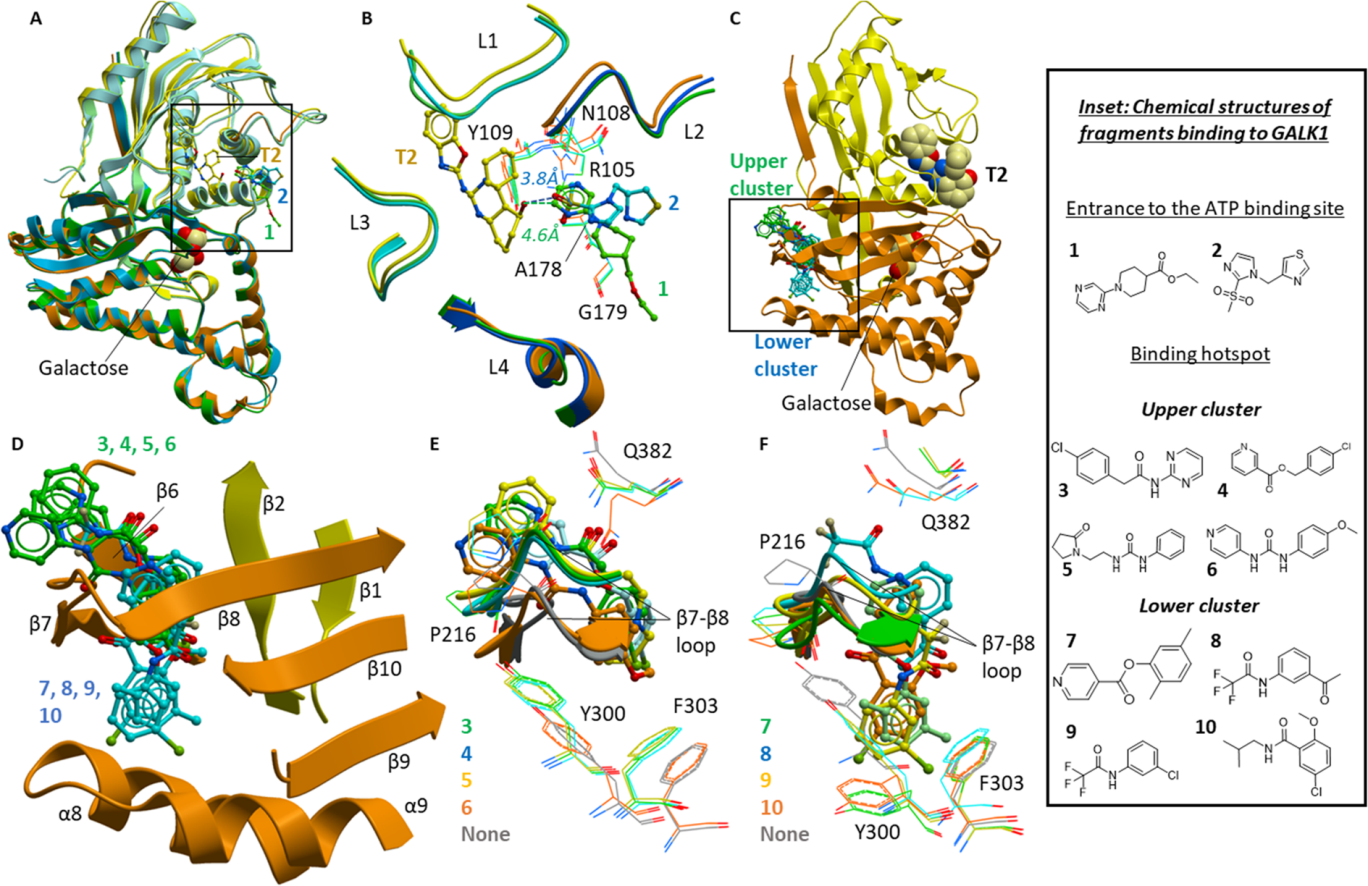
Fragment screening by X-ray crystallography. (A) Superimposed structures of GALK1–galactose–**T2** (yellow/orange), GALK1–galactose–**T2**–fragment **1** (green), and GALK1–galactose–**T2**–fragment **2** (blue). (B) Close-up view of the boxed area shown in panel A, showing the ATP binding loops as a ribbon (L1, Ser79–Pro85; L2, Ser98–Arg105; L3, Val133–Ser140; and L4, Arg228–Glu235), and key residues involved in binding fragments as lines, from structures of GALK1–galactose–**T2** (yellow/orange), GALK1–galactose–**T2**–fragment **1** (light/dark green) and GALK1–galactose–**T2**–fragment **2** (light/dark blue). L2 and L4 are shown in darker shades, and galactose is omitted for clarity. Distances between the **T2** carbonyl and the nearest atoms of fragment **1** (green) or fragment **2** (blue) are indicated by dashed lines. (C) Ribbon diagram of GALK1–galactose–**T2** structure showing the position of fragments in the binding hotspot. Galactose and **T2** are shown as spacefill, fragments from the upper cluster are shown as green sticks, and fragments from the lower cluster are shown as blue sticks. (D) Close-up view of boxed area from panel C, illustrating secondary structure features of the binding hotspot. Fragments from the upper cluster are shown as green sticks, and fragments from the lower cluster are shown as blue sticks. (E and F) Close-up views showing the binding modes of fragments from the upper cluster (E) and lower cluster (F). The β7−β8 loop is shown as ribbon representation, key residues mentioned in the text are shown as lines (color-coded according to the fragment-bound structures), and fragments are shown as sticks.

Fragment-soaked crystals were subjected to high-throughput X-ray data collection and structure determination, to identify bound fragments (Table S2).^37,38^ We observed fragments **1** and **2** bound at the entrance to the active site ATP binding pocket (Figure 2A, Table S2), positioned within 5 Å of the **T2** carbonyl group (Figure 2B). The two fragments do not share a common scaffold (Figure 2, inset), but they both interact with Tyr109, Ala178, and Gly179, as well as compound **T2** (Figure 2B). Fragment **1** forms additional interactions with Asn108, while fragment **2** is close to the backbone of Ala178. The binding of these fragments also causes small movements in Arg105 (Figure 2B), which has been proposed to be essential in hGALK1 activity as described above. The utility of fragments **1** and **2** could be for merging with the larger spiro-benzoxazole inhibitors at the ATP binding site. Relevant to this, the carbonyl group of compound **T2** is 3.2 Å from the proximal oxygen and 3.5 Å from the methyl of the sulfone group of fragment **2** and is 3.6 Å from the proximal nitrogen of the pyrimidine group of fragment **1** (Figure 2B, dotted lines). Therefore, there is potential for linking of these fragments to compound **T2** to expand protein–ligand interactions and improve potency.

### hGALK1-Bound Fragments at an Allosteric Hotspot

Several fragments occupy a hotspot distal from the active site ATP/galactose pockets, on the opposite face of the protein (Figure 2C). This hotspot has not been described for any member of the GHMP kinase family and as such represents a novel pocket of unknown significance. The hotspot is a hydrophobic pocket shielded from bulk solvent by the β7−β8 loop (Pro212–Leu218). The pocket is lined on one side by strands β8– β10 (Leu218–Thr223, Tyr339–Met343 and Gly350–Ala358) and on the other side by the loop preceding β6 (Gly193–Gly196) and strands β6 and β7 (His197–Asp202 and Thr208–Val211; Figure 2D). Fragments binding in this hotspot pocket can be classified into upper (fragments **3**–**6**, green sticks in Figure 2C–D; Table S3) and lower (fragments **7**–**10**, blue sticks in Figure 2C,D; Table S4) clusters, according to whether they extend above or below the β7−β8 loop.

Fragments from the upper cluster consist of an aromatic ring separated by an amide, ester, or urea linker from another ring, which is usually aromatic (Figure 2, inset). One of the two constituent rings occupy the center of the pocket, stabilized by hydrophobic interactions with several residues, including Tyr300 and Phe303, which are displaced inward to do so (Figure 2E). The linker of each fragment hydrogen bonds with residues of the β7−β8 loop and the remaining, heterocyclic, ring is more surface exposed and interacts with the side chains of Pro216 and Gln382. Fragments from the lower cluster are smaller in size; they contain one ring occupying the center of the pocket as with upper cluster fragments and an additional variable group that extends below the β7−β8 loop, displacing the Tyr300 and Phe303 side chains to avoid steric clashes (Figure 2F).

### Fragments Inhibit hGALK1 Activity without Competing with ATP or Galactose

**Figure 3:**
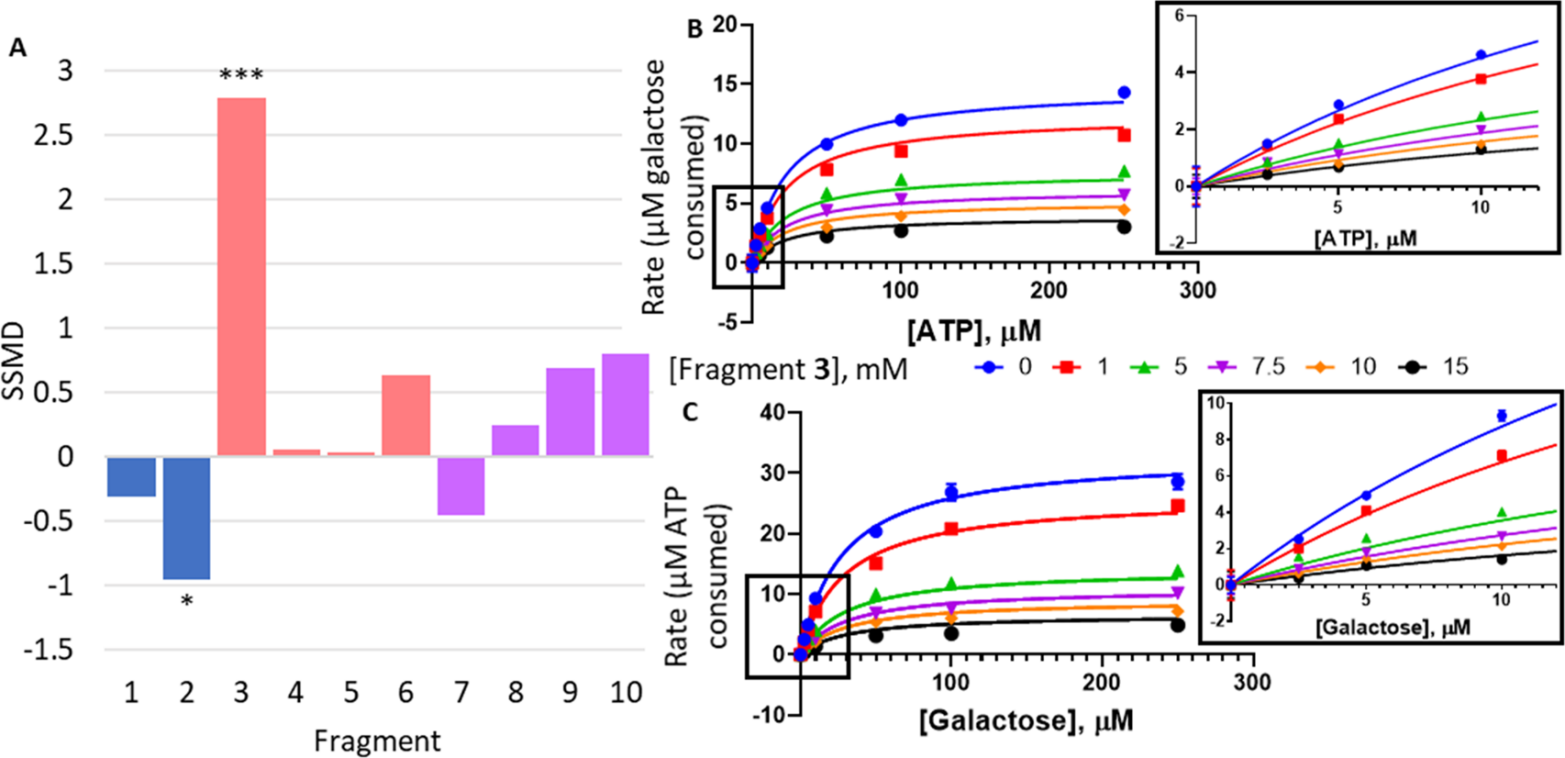
Change in hGALK1 activity in the presence of fragments. (A) Bar chart showing the observed change in hGALK1 activity in the presence of 5 mM fragment, as measured in the Kinase-Glo assay. Change in activity is reported as strictly standardized mean difference (SSMD), and *P* values are also indicated: *P < 0.05, ***P < 0.0001. (B) Least-squares nonlinear fit of GALK1 reaction rate (total galactose consumed after 1 h reaction, μM) against increasing ATP concentrations (0–250 μM) in the presence of different concentrations of fragment **3** (0–15 mM). Curves were fitted to a noncompetitive inhibition model, the best fitting enzyme kinetics–inhibition equation, using the GraphPad Prism software. Inset: Close-up view of plot showing GALK1 reaction rate (total galactose consumed after 1 h reaction, μM) against increasing ATP concentrations (0–10 μM) in the presence of different concentrations of fragment **3** (0–15 mM), as determined in the Amplex Red assay. (C) Least-squares nonlinear fit of GALK1 reaction rate (total ATP consumed after 1 h reaction, μM) against increasing galactose concentrations (0–250 μM) in the presence of different concentrations of fragment **3** (0–15 mM). Curves were fitted to a noncompetitive inhibition model, the best fitting enzyme kinetics–inhibition equation, using the GraphPad Prism software. Inset: Close-up view of plot showing GALK1 reaction rate (total ATP consumed after 1 h reaction, μM) against increasing galactose concentrations (0–10 μM) in the presence of different concentrations of fragment **3** (0–15 mM), as determined in the Kinase-Glo assay.

We first determined the inhibitory effects of fragments on hGALK1 activity using the Kinase-Glo assay. Single-point profiling of fragments revealed weak inhibition for fragment **3** from the upper hotspot cluster, reducing hGALK1 activity by 24 ± 1.6% at 5 mM fragment, an effect that is statistically significant (*P* < 0.0001; Strictly Standardized Mean Difference, SSMD, = 2.79; Figure 3A).

For other fragments tested, either there is no observed inhibition (i.e., SSMD < 0.5; fragments **1**, **2**, **4**, **5**, **7**, and **8**) or their inhibitory effects were not statistically significant (fragments **6**, SSMD = 0.69; **9**, SSMD = 0.80; and **10**, SSMD = 0.80; *P* > 0.05 in all cases). The lack of inhibition by fragments **1** and **2** at the active site entrance suggest that these fragments are only bound in the structure due to the proximity of **T2** present in the crystal system.

We next investigated whether the inhibitory effect of fragment **3** is mediated by its binding to the active site (akin to the spiro-benzoxazole inhibitors) or a nonorthosteric site (such as that revealed from its costructure). Galactose-titration experiments with fragment **3** showed a concentration-dependent, 5-fold decrease in Vmax_app_ (*P* < 0.0001) and a 6-fold increase in *K*m_app_ (*P* < 0.0001), collectively indicating mixed model inhibition with galactose by fragment **3**, which is supported by fitting in GraphPad Prism (Figure 3C and Table S5).

Additionally, ATP-titration experiments with fragment **3** showed a concentration-dependent, 4-fold decrease in Vmax_app_ (*P* < 0.0001) without change in the *K*m_app_ (*P* = 0.4932), collectively indicating noncompetitive inhibition with ATP by fragment **3**, which is supported by fitting in GraphPad Prism (Figure 3B and Table S5). Collectively, these results suggest that fragment **3** does not bind to the active site of hGALK1, consistent with its costructure, and that the hotspot is a true allosteric binding site.

### Optimizing Hotspot Fragments into a Micromolar Selective Inhibitor

**Figure 4:**
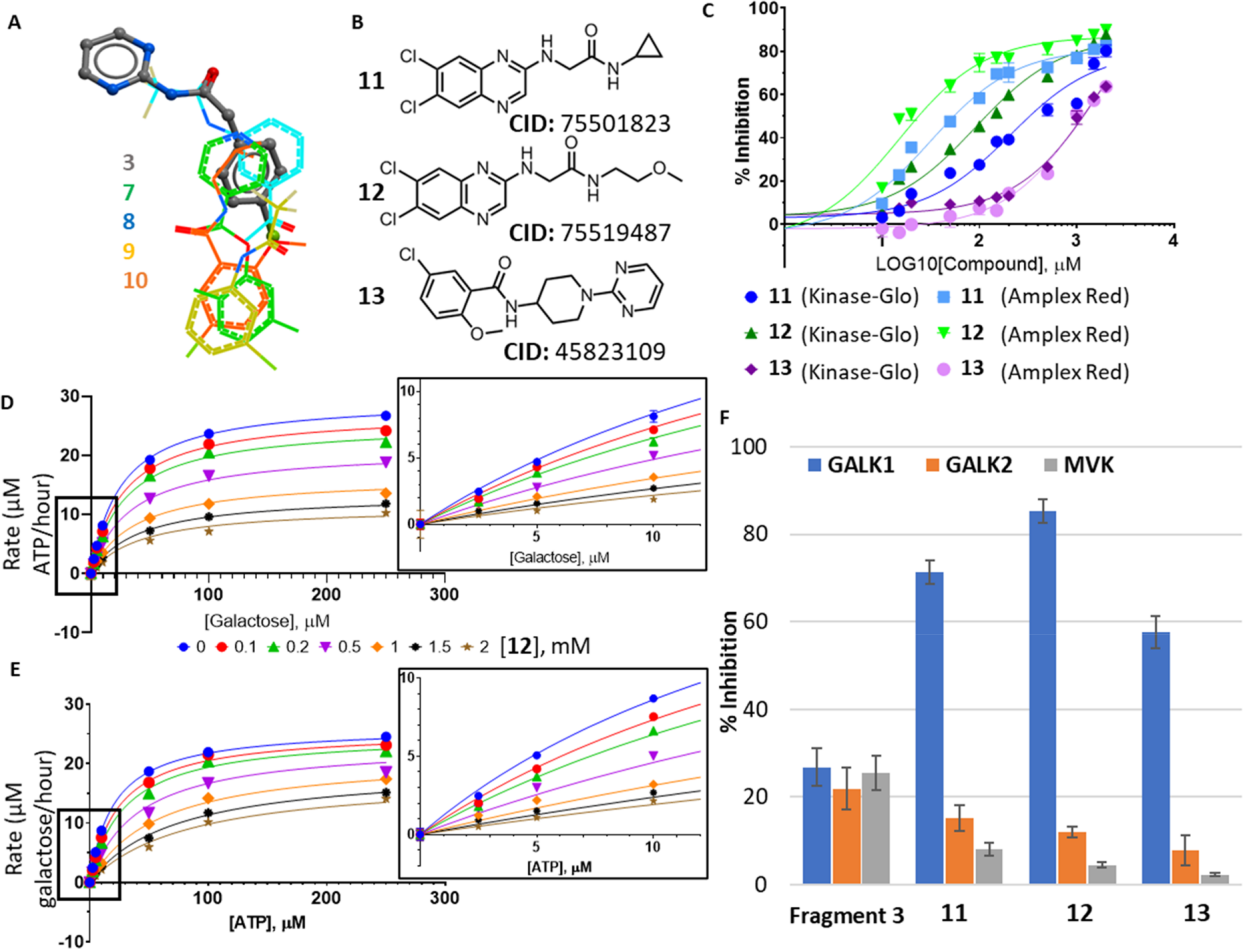
Follow-up compounds based on hotspot fragments. (A) Ligand view of superimposed fragment structures at the binding hotspot. Fragment **3** from the upper cluster is shown as sticks, and fragments **7**–**10** from the lower cluster are shown as lines. (B) Chemical structures and chemical IDs of follow-up compounds **11**–**13**. (C) Concentration–response curves for compounds **11** (blue), **12** (green), and **13** (purple) as measured in the Kinase-Glo assay (dark shades) and the Amplex Red assay (light shades). (D) Least-squares nonlinear fit of GALK1 reaction rate (total ATP consumed after 1 h reaction, μM) against increasing galactose concentrations (0–250 μM) in the presence of different concentrations of **12** (0–2 mM). Curves were fitted to a noncompetitive inhibition model, the best fitting enzyme kinetics–inhibition equation, in GraphPad Prism. Inset: Close-up view of plot showing GALK1 reaction rate (total ATP consumed after 1 h reaction, μM) against increasing galactose concentrations (0–10 μM) in the presence of different concentrations of **12** (0–2 mM), as determined in the Kinase-Glo assay. (E) Least-squares nonlinear fit of GALK1 reaction rate (total galactose consumed after 1 h reaction, μM) against increasing ATP concentrations (0–250 μM) in the presence of different concentrations of **12** (0–2 mM). Curves were fitted to noncompetitive inhibition model, the best fitting enzyme kinetics–inhibition equation, in GraphPad Prism. Inset: Close-up view of plot showing GALK1 reaction rate (total galactose consumed after 1 h reaction, μM) against increasing ATP concentrations (0–10 μM) in the presence of different concentrations of **12** (0–2 mM), as determined in the Amplex Red assay. (F) Bar chart comparing % inhibition of hGALK1 (blue), hGALK2 (orange), and hMVK (gray) by 5 mM of fragment **3** or 1 mM of **11**–**13**.

Fragments provide a tractable path for optimization into potent compounds through approaches of fragment growing, linking, and merging.^39^ We overlaid fragment **3** from the upper cluster and fragments from the lower cluster in the costructures and searched commercial catalogues for compounds that incorporate chemical groups from both fragment clusters (Figure 4A; see Supporting Information for more details), with the expectation that such compounds could engage with both upper and lower components of the hotspot. We procured three compounds **11**–**13** (Figure 4B) of molecular weights 300–500 Da.

Compounds **11**–**13** demonstrated >50% inhibition of hGALK1 at 2 mM concentration in both Kinase-Glo (Figure S4A) and Amplex Red assays (Figure S4B). Dose-dependent sigmoidal curves (Figure 4C) reveal IC_50_ values in the range of 31–209 μM for **11** (Astex Therapeutics ligand lipophilicity efficiency score, LLE_AT_, 0.19–0.22 kcal/mol) and 25–198 μM for **12** (LLE_AT_ 0.23–0.27 kcal/mol) (Table S6). Therefore, **11** and **12**, bearing a 6,7-dichloro-quinoxaline scaffold, share similar IC_50_ values, with **12** achieving a slightly higher ligand efficiency. Determination of IC_50_ values was not possible for **13** due to its lower potency, with 50% inhibition achieved by approximately 1 mM of **13** in both assays.

The modes of inhibition for **11**–**13** with respect to galactose and ATP were then determined by comparison of nonlinear least-squares fitting to inhibition equations as described for fragment **3**. Compounds **11** and **12** were noncompetitive with respect to both ATP and galactose, whereas **13** demonstrated mixed inhibition with respect to galactose and noncompetitive inhibition with respect to ATP [Figures 4D,E (compound **12**), S5, and S6 (compounds **11** and **13**); Table S5 (compounds **11**–**13**)]. Altogether, our kinetics data indicate that inhibition of hGALK1 activity by fragment **3** and follow-up compounds **11**–**13** is consistent with their binding away from the active site, in agreement with the fragment-bound crystal structures.

**Figure 5:**
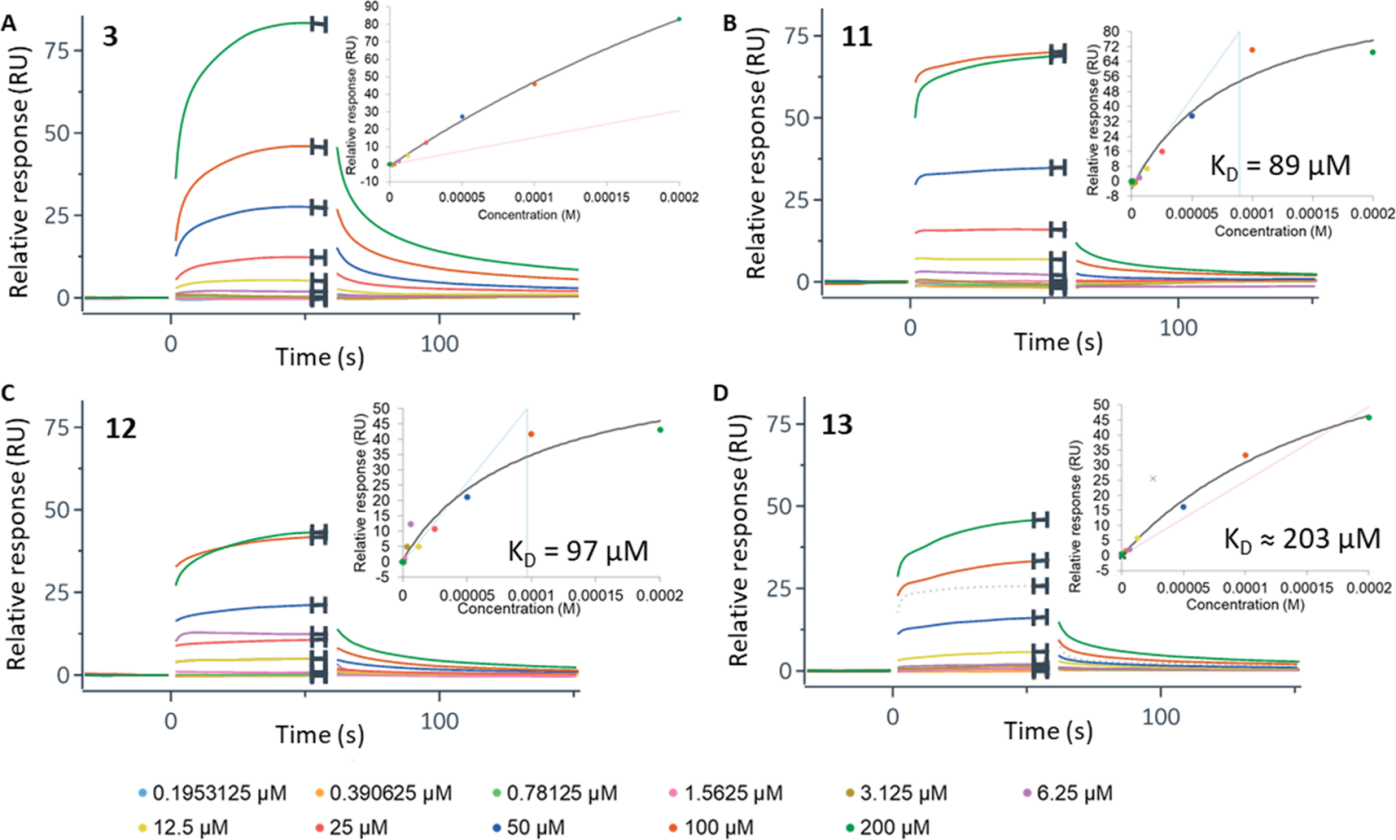
Characterization of fragment **3** and compounds **11**–**13** binding to hGALK1 by SPR. (A–D) Sensorgram plots of relative response, in response units (RU), against time for different concentrations of fragment **3** (A), compound **11** (B), compound **12** (C), and compound **13** (D). Curves are colored according to ligand concentration, as defined in the key. Inset: Sensorgram plots of relative response against ligand concentration, extracted from the plots of relative response against time. *K*_D_ value for fragment **3** cannot be reliably determined.

To further confirm binding of fragment **3** and follow-up compounds **11**–**13** to hGALK1 in solution, we performed surface plasmon resonance (SPR) measuring dissociation constants (*K*_D_) of each compound upon binding to hGALK1 covalently immobilized on a sensor chip. Weak binding was observed for fragment **3**, with *K*_D_ in the high-micromolar to low-micromolar range (Figure 5A). Consistent with the activity assay results (Figure 4; Results and Discussion section), compounds **11** (Figure 5B) and **12** (Figure 5C) demonstrated similar binding efficiency with *K*_D_ values around 100 μM, while compound **13** (Figure 5D) showed a slightly weaker *K*_D_ of around 200 μM.

*In silico* docking indicates that compounds **11**–**13** can bind to the hotspot pocket, interacting with the same set of residues that are involved in binding fragments (Supplementary Results; Table S6-9; Figure S7). For example, predicted docking poses of **11** (Figure S7B) and **12** (Figure S7C) show the 6,7-dichloro-quinoxaline group occupying the lower binding pocket while the flexible group interacts with surface loops around the upper binding pocket. Predicted docking poses of **13** (Figure S7D) indicate this compound interacts more with the upper binding pocket but forms fewer interactions with the lower binding pocket.

GALK1 is one of several small-molecule kinases from the GHMP class. We measured the activity of two other human GHMP kinases, N-acetyl galactosamine kinase (hGALK2, 32.5% sequence identity to hGALK1; EC 2.7.1.157) and mevalonate kinase (hMVK, 26.2% sequence identity; EC 2.7.1.36), in the presence of fragment **3** or compounds **11**–**13**, to determine the relative selectivity of these inhibitors. While fragment **3** demonstrates a similar degree of inhibition against hGALK1, hGALK2, and hMVK, compounds **11**–**13** by contrast were selective for hGALK1, displaying less than 10% inhibition of hGALK2 and hMVK at 1 mM (Figure 4E). This selectivity can be rationalized by the poor structural conservation of the nonorthosteric fragment hotspot among GHMP members, particularly in the equivalent regions of the β7−β8 loop that forms the front of the hotspot and the α8–turn−α9 segment that forms the base of the hotspot (Figure S8). At the sequence level, these two regions are also poorly conserved (Figure S9). Finally, the β7−β8 loop and the α8−α9 turn differ in conformation among all hGALK1 protomers within the asymmetric unit, indicating conformational plasticity in this region that can be modulated by ligand binding (Figure S10).

### Concluding Remarks

This work reveals chemical starting points bound to a non-orthosteric hotspot region, which can be exploited for future development of hGALK1-specific allosteric inhibitors. Allosteric inhibitors often promote structural rearrangements to an enzyme’s active site that can decrease the enzyme’s affinity for its substrates, affect the formation of the enzyme–substrate complex, or change the stability of the transition state. In hGALK1, molecular dynamics studies have pinpointed Arg105 in the active site and two regions (residues 174–179 and 231–240), where conformational flexibility is important for catalysis.^40^ Future MD and structural studies are therefore merited to investigate whether these regions, or others, are involved in a long-range allosteric communication for hGALK1, to guide further development of small molecules that can target such mechanism(s) for higher potency.

## Materials and Methods

### Chemicals

Compounds **T1** and **T2** were purchased from Interbioscreen. All fragments were purchased from Enamine.

### Expression and Purification of hGALK1

A hGALK1 construct, encoding full-length protein harboring the surface entropy^41^ mutations K252A:E253A, with an engineered N-terminal His6-tag subcloned into the pET21d vector, was transformed into *E. coli* BL21(DE3) cells. hGALK1 was cultured in Terrific Broth with 0.1 mM IPTG induction at 18 °C. Cell pellets were harvested, homogenized in lysis buffer (50 mM sodium phosphate pH 7.4, 500 mM NaCl, 5% glycerol, 0.5 mM TCEP, 30 mM galactose), and centrifuged to remove insoluble material. The supernatant was purified by nickel affinity (Thermo Fisher Scientific) followed by size exclusion (Superdex 200 Hi-Load 16/60, GE Healthcare) chromatography into crystallization buffer (50 mM sodium phosphate pH 7.4, 500 mM NaCl, 5% glycerol, 30 mM Galactose and 0.5 mM TCEP). Purified protein was concentrated to 24 mg mL^–1^. Constructs encoding full-length hGALK2 (Met1-Ala458) and hMVK (Met1-Leu396) were subcloned into the pNIC-NHStIIT vector encoding an engineered N-terminal His6–Strep–Strep-tag followed by a TEV protease cleavage site. Each construct was expressed and purified as described for hGALK1.

### Co-crystallization with Spiro-benzoxazole Inhibitors

To co-crystallize hGALK1 with galactose and 2′-(benzo[*d*]oxazol-2-ylamino)-7′,8′-dihydro-1′*H*-spiro[cyclopentane-1,4′-quinazolin]-5′(6′*H*)-one (**T1**, compound entry 3 in^25^), 24 mg mL^–1^ of purified hGALK1 was preincubated with 5 mM **T1** dissolved in *N*-methylpyrrolidone (NMP; final concentration 10%), and crystals were grown by sitting-drop vapor diffusion at 20 °C, equilibrated against a well solution of 0.1 M Morpheus Amino Acids Mix, 0.1 M Morpheus Buffer system 1 pH 6.5, and 50% v/v Morpheus Precipitant Mix 1. To co-crystallize hGALK1 with galactose and 2′-(benzo[*d*]oxazol-2-ylamino)-7′,8′-dihydro-1′*H*-spiro[cyclohexane-1,4′-quinazolin]-5′(6′*H*)-one (**T2**, compound entry 4 in^25^), 24 mg mL^–1^ of purified GALK1 was preincubated with 2.25 mM **T2** dissolved in NMP (final concentration 5%) and crystals grown by sitting-drop vapor diffusion at 20 °C, equilibrated against a well solution of 0.1 M Morpheus Carboxylic Acids Mix, 0.1 M Morpheus Buffer system 2 pH 7.5, and 50% v/v Morpheus Precipitant Mix 4. Crystals were cryo-protected with 50% NMP and flash cooled in liquid nitrogen. Diffraction data were collected at Diamond Light Source beamline I04 and indexed, integrated, and scaled via the automated Xia2 pipeline.^42^ Molecular replacement, using the hGALK1–galactose–AMPPNP structure (PDB 1WUU) as a search template, and subsequent model building and refinement were performed in the CCP4 program suite.^43^

### Crystallography-Based Fragment Screening

Purified hGALK1 at 24 mg mL^–1^ was preincubated with 2.25 mM of compound **T2** dissolved in NMP (final concentration 5%). Hundreds of crystals were mass-produced by sitting-drop vapor diffusion at 20 °C, equilibrated against well solutions of 0.1 M MOPS/sodium HEPES pH 7.0–8.0 (based on Morpheus Buffer system 2), 40–50% Morpheus Precipitant Mix 4, and 0.1 M Morpheus Carboxylic acids mix. For soaking, 50 nL of each fragment (∼200 fragments from the DSi-Poised Library;^26^ from supersaturated stock solutions of 100–500 mM in d6-DMSO, resulting in final concentration of 25–125 mM fragment) was added to a crystallization drop using an ECHO acoustic liquid handler dispenser at the Diamond Light Source beamline I04-1. Crystals were soaked for 2 h with fragments (final concentration of 25–125 mM) before being harvested using the SHIFTER technology,^44^ cryo-cooled in liquid nitrogen, and measured using the “automated unattended” mode of the i04-1 beamline. The XChemExplorer pipeline^45^ was used for structure solution with parallel molecular replacement using DIMPLE,^46^ followed by map averaging and statistical modeling to identify weak electron densities generated from low occupancy fragments using PanDDA.^47^ Model building and refinement were performed using the WinCoot and REFMAC software integrated into the XChemExplorer pipeline.^45^ Figures were prepared using ICM-Pro software (Molsoft LLC).

### Kinase Glo Activity Assay

hGALK1 activity *in vitro* was determined using the Kinase-Glo luminescent kinase assay (Promega), according to the manufacturer’s protocol. Determination of suitable assay parameters is described in the Supplementary Methods and shown in Figure S1. To measure activity and inhibition of hGALK1, 10 μL/well of reaction containing 10 nM hGALK1, 100 μM galactose, and 35 μM ATP in an assay buffer (50 mM sodium phosphate pH 7, 200 mM potassium chloride, 20 mM magnesium chloride, 0.01% Triton-X) was incubated with 5 mM of fragments; varying concentrations of spiro-benzoxazole compounds **T1** and **T2** (0–1 mM, 12 concentrations) or compounds **11**–**13** (0–2 mM, 12 concentrations) were dispensed into 384-well assay plates (Greiner). Similarly, hGALK2 activity and inhibition was measured with reactions containing 50 nM hGALK2, 100 μM N-acetylgalactosamine, and 35 μM ATP in the above-described buffer and hMVK activity and inhibition was measured with reactions containing 250 nM hMVK, 100 μM mevalonate, and 35 μM ATP in the above-described buffer. Following a 1-h RT incubation, 10 μL of Kinase-Glo Plus detection reagent was added (final assay volume: 20 μL/well), and after a further 20 minutes RT incubation, luminescence was detected using a Pherastar FSX plate reader (BMG Labtech) containing a luminescence optics module. To determine the inhibition mode with respect to galactose, 10 nM hGALK1 and 35 μM ATP in an assay buffer was added to an array of six fragment (0–15 mM) or 12 compound (0–2 mM) concentrations against six galactose concentrations (0–250 μM), with a final reaction volume of 10 μL/well dispensed into 384-well assay plates (Greiner), and measured as described above. All reactions were performed in technical triplicates for two different preparations of hGALK1. Reaction rate, defined as total ATP consumed over the 1-h hGALK1 reaction, was determined using the following equation, where Lum_reaction_ = mean value of measured luminescence of each reaction and Lum_35_ = measured luminescence with no galactose (i.e., luminescence of 35 μM ATP):
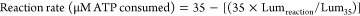


Data were plotted using GraphPad Prism software; curve fitting was performed with a nonlinear least-squares regression fit to the mixed, competitive, noncompetitive, and uncompetitive inhibition models from the GraphPad Prism enzyme kinetics–inhibition equations and the best fit selected by comparison of Akaike’s Information Criterion (AICc) probability scores and extra sum-of-squares *F* test *P* values calculated by the software.

### Amplex Red Activity Assay

The Amplex Red assay reagent contained horseradish peroxidase (EC 1.11.1.7, 0.2 U/mL), its substrate Amplex Red (10-acetyl-3,7-dihydroxyphenoxazine, 100 μM), and galactose oxidase (EC 1.1.3.9, 4 U/mL). The assay buffer used was the same as in the Kinase-Glo assay. Determination of suitable assay parameters is described in the Supplementary Methods and shown in Figure S3. The reaction conditions were 250 nM hGALK1, 50 μM galactose, and 100 μM ATP for IC_50_ calculation and 250 nM hGALK1, 50 μM galactose, and 0–250 μM ATP for determination of inhibition mode. Amplex Red reagent was added at a 1:1 volume to the hGALK1 reaction after 1-h incubation at RT. Fluorescence emission was measured at 585 nm, with excitation at 570 nm, after a further incubation period of 40 minutes, using a Pherastar FSX plate reader with a FI 540 590 optics module. Reactions were performed in technical triplicates for two biological replicates, and data were plotted and fitted as described for the Kinase-Glo assay except that the reaction rate, defined as total galactose consumed over the 1-h hGALK1 reaction, was determined using the following equation, where Fluor_reaction_ = mean value of measured fluorescence of each reaction and Fluor_50_ = measured fluorescence with no galactose (i.e., fluorescence of 50 μM galactose):
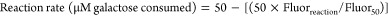


### Surface Plasmon Resonance (SPR) Binding Assay

Purified hGALK1 (30 μg/mL), diluted in acetate buffer at pH 5, was attached via covalent coupling to a density of 13000 RU on a CM5 chip. The assay buffer was 50 mM sodium phosphate, pH 7.5, 200 mM NaCl, 0.5 mM TCEP, 5% DMSO, and 0.05% TWEEN20. A serial dilution (11 concentrations) was prepared in the above buffer for each analyte (**3**, **11**–**13**) by 1:1 dilution from 200 μM to 0.195 μM, and the subsequent solutions were passed over the chip at a flow rate of 30 μL/min. Data are from an *n* = 1 experiment.

## Supporting Information Available

Methods, results, and figures relating to the setup and optimization of activity assays; the design, docking, and characterization of compounds **11**–**13**; refinement statistics and superimposition of reported crystal structures; and the alignment of GHMP kinase sequences and structures (PDF)
