# Supplementary material for: Fragment Screening Reveals Starting Points for Rational Design of Galactokinase 1 Inhibitors to Treat Classic Galactosemia
Source: ACS Chem Biol. 2021 Mar 16;16(4):586–95. doi: 10.1021/acschembio.0c00498 (PMC8056384; doi:10.1021/acschembio.0c00498)
Supplement: Supplementary file 1 — cb0c00498_si_001.pdf [file cb0c00498_si_001.pdf]

Supporting Information

Fragment screening reveals starting points for rational design of galactokinase 1 inhibitors to treat classic galactosemia

Sabrina R. Mackinnon[†‖], Tobias Krojer[†], William R. Foster[†], Laura Diaz-Saez[†, ‖], Manshu Tang[‡], Kilian V.M. Huber[†, ‖], Frank von Delft[†, §], Kent Lai[‡], Paul E. Brennan[†, ‖*], Gustavo Arruda Bezerra[*,†], Wyatt W. Yue[*,†]

[1]Structural Genomics Consortium, Nuffield Department of Medicine, University of Oxford, Oxford, United Kingdom, OX3 7DQ
[‡]Department of Paediatrics, University of Utah, Salt Lake City, UT84108, United States
[§]Diamond Light Source, Harwell Science and Innovation Campus, Didcot, Oxfordshire, U.K.
[‖]Target Discovery Institute, University of Oxford, United Kingdom, OX3 7FZ

*Corresponding authors:
Wyatt W. Yue wyatt.yue@cmd.ox.ac.uk
Gustavo A. Bezerra gustavo.arrudabezerra@cmd.ox.ac.uk
Paul E. Brennan paul.brennan@ndm.ox.ac.uk

Supplementary Methods 2

Supplementary Results 4

Supplementary Tables S1 – S9 6

Supplementary Figures S1 – S11 13

Supplementary methods

Drawing of theoretical follow-up compounds. Datasets of hGALK1 co-structures with fragments bound at the binding hotspot (fragments **3** – **10**) were superimposed using ICM-Pro software and pairs of upper (**3** – **6**) and lower (**7** – **10**) cluster fragments were visually inspected to identify overlapping motifs (Figure S11A). Theoretical follow-up compounds, comprising functional groups from both upper and lower cluster fragments, were then drawn using ChemDraw software (Figure S11B).

Search for commercially available follow-up compounds. For each drawn compound, a similarity search of the Enamine Screening Compounds library was performed with a threshold of 0.8. Three representative compounds (**11** – **13**; Figure S11C) were selected from the search results for testing in solution.

*Docking of compounds **11-13**.* Docking was performed in ICM-Pro software (Molsoft LLC). To prepare the docking receptor, the hGALK1 structures solved in this work and the previously published hGALK1-galactose-AMPPNP structure (PDB code 1wuu) were converted to ICM objects, minimising hydrogens and deleting waters. After global energy minimisation, each protein chain was split into a separate object, ligands in the binding hotspot were removed and all chains were superimposed. Next, the receptor maps were made using the ICM 4D docking function, incorporating all protein chains to produce a receptor ensemble, with the binding pocket defined as residues within 5 Å of any fragment. Each compound was then converted to 3D and minimised before docking into the prepared receptor. An ICM score cut-off of -20 (with a lower score indicating a better predicted interaction) was applied to filter the resulting docking poses. The ICM docking score is a combined metric incorporating: ligand strain (kcal/mol), van der Waals interaction energy, number of torsions in the ligand, the solvation electrostatics energy change upon binding, the hydrogen bond energy, hydrophobic energy in exposing a surface to water, and the desolvation of exposed H-bond donors and acceptors.

Determination of suitable hGALK1 concentration for activity assays. Linearity of the hGALK1 reaction, as measured by the Kinase-Glo assay, was assessed for 10 nM, 50 nM, 250 nM and 500 nM hGALK1 by addition of 50 µM ATP to triplicate reactions containing hGALK1 and 100 µM galactose. A new reaction was started every 2 minutes for 92 minutes. After 92 minutes, the Kinase-Glo reagent was added to stop the hGALK1 reaction and luminescence was measured after a further 20 minutes incubation. Two additional reactions, one containing hGALK1 but not galactose and one containing hGALK1 and galactose but not ATP were

included as controls. All reactions were performed in 10 µL reaction volumes and an equal volume of Kinase-Glo reagent was added. Data were analysed by plotting the product concentration (ATP consumed, µM, calculated as described in the main text) against time (minutes incubated with hGALK1 before addition of Kinase-Glo reagent) followed by least squares regression fit to the Michaelis-Menten equation using GraphPad Prism software (Figure S1B). Similar experiments were performed to assess linearity of the hGALK1 reaction in the Amplex Red assay for the same concentrations of hGALK1 over the same time period. Specifically, reactions containing hGALK1 and 100 µM ATP were started every 2 minutes for 92 minutes by addition of 50 µM galactose, reactions were stopped by addition of an equal volume of Amplex Red reagent and fluorescence (570 nm excitation, 585 nm emission) was measured after a further 40 minutes incubation. Control samples lacking either galactose or ATP were also included. Data were analysed by plotting the product concentration (galactose consumed, µM, calculated as described in the main text) against time (minutes incubated with hGALK1 before addition of Amplex Red reagent) followed by non-linear least squares fit to the Michaelis-Menten equation using GraphPad Prism software (Figure S3B).

Determination of initial substrate concentration for activity assays. The linear range for both assays was determined by measuring the appropriate signal upon adding the detection reagent to a serial dilution (from 200 µM) of the substrate to be measured and analysed by least squares linear regression in GraphPad Prism i.e. standard curves of luminescence against ATP concentration (Figure S1C) and of fluorescence against galactose concentrations (Figure S3C) were plotted.

Determination of Michaelis-Menten constants (Km_{app}) in activity assays. The Km_{app} for galactose was measured in the Kinase-Glo assay by incubation of 10 nM hGALK1, 35 µM ATP and an array of galactose concentrations (0 – 1 mM) for 1-hour (10 µL/well) before addition of an equal volume of Kinase-Glo reagent, further incubation for 20 minutes, and measurement of luminescence signal. Data were plotted in GraphPad Prism and analysed by non-linear least squares fit to the Michaelis-Menten equation using GraphPad Prism software (Figure S1D). The Km_{app} for ATP was measured in the Amplex Red assay by incubation of 250 nM hGALK1, 50 µM galactose and an array of ATP concentrations (0 – 0.5 mM) for 1-hour (10 µL/well) before addition of an equal volume of Amplex Red reagent, further incubation for 40 minutes, and measurement of fluorescence signal (excitation 570 nm, emission 585 nm). Data were plotted in GraphPad Prism and analysed by non-linear least squares fit to the Michaelis-Menten equation using GraphPad Prism software (Figure S3D).

Supplementary Results

Determination of suitable activity assay parameters. hGALK1 activity was measured at different protein concentrations to determine a protein concentration that yielded sufficient signal and for which the signal was linear after 60 minutes reaction time. For the Kinase-Glo assay, this was 10 nM hGALK1 (blue, Figure S1B) and for the Amplex Red assay, this was 250 nM hGALK1 (green, Figure S3B). A suitable concentration of the constant substrate (ATP in Kinase-Glo and galactose in Amplex Red) was selected by plotting standard curves of the substrate against the reaction rate (defined as the amount of constant substrate consumed per minute of the 1-hour reaction; μM/min). After analysis of the resulting standard curve for the Kinase-Glo assay (Figure S1C) and the Amplex Red assay (Figure S3C), the concentration of the constant substrate was selected to be 35 μM ATP and 50 μM galactose. The Kinase-Glo assay was used to determine the $Vmax_{app}$ (0.36 ± 0.03 μM/min) and $Km_{Gal, app}$ (54.6 ± 16.9 μM) for 10 nM hGALK1 in the presence of 35 μM ATP (Figure S1D). Similarly, the Amplex Red assay was used to determine the $Vmax_{app}$ (0.5 ± 0.05 μM/min) and $Km_{ATP, app}$ (43.0 ± 7.9 μM) for 250 nM hGALK1 in the presence of 50 μM galactose (Figure S3D).

Docking of compounds 11-13. To account for the conformational plasticity observed at the binding hotspot pocket, an ensemble of all hGALK1 structures was employed as the docking receptor. Docking of compounds **11-13** to the hGALK1 ensemble resulted in 10, 14 and 8 conformations above the selected score cut-off (ICM Score < -20; lilac, yellow and blue sticks in Figure S7A). The 6,7-dichloro-quinoxaline scaffold of compounds **11** and **12** occupies a similar position in the binding hotspot as the lower cluster of fragments in all but one ranked compound **11** poses (Figure S7B) and all ranked compound **12** poses (Figure S7C). In the docked poses of compound **13** this position is occupied by the smaller chloro-methoxybenzyl group (Figure S7D). The N-cyclopropylacetamide group of compound **11** and the N-(2-methoxyethyl)acetamide group of compound **12** both show two possible poses (Figure S7B and S7C respectively). One conformation occupies a similar space to the upper cluster of fragments, interacting primarily with residues of the β7-β8 loop and the other conformation is at a 180° angle to the first, interacting with residues of the β7-β8 loop and also the β11-β12 loop (yellow ribbon in Figure S7B-D). Compound **11** favours the former position, reflected by the occurrence of the three highest scored poses in this conformation, while compound **12**, favours the latter position, with the three highest scored poses clustering to this position. The N-(1-(pyrimidin-2-yl)piperidin-4-yl)acetamide group of compound **13** is only found in the latter conformation, reflecting the less flexible structure of this compound (Figure S7D). The

ICM score and component scoring functions for docking poses of compounds **11-13**, above the selected cut-off, are shown in Tables S7-S9 and Table S10 compares the best result for each function per compound. All three compounds are similar in overall score and are predicted to make similarly favourable van der Waals and hydrophobic interactions. Binding of compound **13** is less favourable in terms of solvation electrostatics, which may account for the weaker activity and binding assay results for this compound.

Table S1: Crystallographic refinement statistics for hGALK1 co-crystal structures with spiro-benzoxazole inhibitors T1 and T2.

	hGALK1-galactose-T1	hGALK1-galactose-T2
**Data collection**		
Beamline	DLS I03	DLS I04
Wavelength (Å)	0.97624	0.97950
Space group	*P* 21	*F* 2 2 2
Unit cell parameters (Å; °)	a=119.9, b=97.2, c=144.9; α=γ=90.0, β=98.5	a=107.7, b=151.7, c=230.6; α=β=γ=90.0
Resolution (Å)	57.9 - 2.5	57.8 - 1.8
Observed/Unique reflections	746619/110543	421458/73958
Multiplicity	6.8 (6.6)[a]	5.7 (3.0)[a]
Completeness	99.1 (98.7)[a]	89.35 (50.0)[a]
I/sig(I)	7.3 (0.9)[a]	11.5 (1.0)[a]
R-merge (%)	20.0 (247.0)[a]	8.0 (64.0)[a]
Correlation CC (1/2)	99.6 (44.4)[a]	99.8 (55.5)[a]
**Refinement statistics**		
Rwork (%)	21.4	19.6
Rfree (%)	21.7	20.2
R.m.s.d. bond length (Å)	0.008	0.008
R.m.s.d. bond angle (°)	1.5	1.2
PDB code	6ZFH	6Q3X

[a] Values in brackets correspond to the highest resolution shell

Table S2: Crystallographic refinement statistics for hGALK1 co-crystal structures with fragments 1 and 2.

	**hGALK1-galactose-T2-1**	**hGALK1-galactose-T2-2**
**Data collection**		
Beamline	DLS I04-1	DLS I04-1
Wavelength (Å)	0.91587	0.91587
Space group	*P* 21	*P* 21
Unit cell parameters (Å, °)	a=73.3, b=114.8, c=120.8; α=γ=90.0, β=100.5	a=73.7, b=114.9, c=121.0; α=γ=90.0, β=100.6
Resolution (Å)	82.6 - 1.9	82.7 - 2.4
Observed/Unique reflections	472391/ 139631	264639/77146
Multiplicity	3.4 (3.5)[a]	3.4 (3.5)[a]
Completeness	99.3 (99.5)[a]	99.5 (99.5)[a]
I/sig(I)	7.2 (0.8)[a]	11.7 (2.2)[a]
R-merge (%)	11.7 (195.7)[a]	6.4 (47.1)[a]
CC (1/2) (%)	99.6 (38.7)[a]	99.8 (89.4)[a]
**Refinement statistics**		
Rwork (%)	21.4	19.6
Rfree (%)	21.7	20.2
R.m.s.d. bond length (Å)	0.008	0.008
R.m.s.d. bond angle (°)	1.5	1.2
PDB code	6Q3W	6QJE

[a] Values in brackets correspond to the highest resolution shell

Table S3: Crystallographic refinement statistics for hGALK1 co-crystal structures with fragments 3 – 6, binding in the allosteric hotspot.

	**hGALK1-galactose-T2-3**	**hGALK1-galactose-T2-4**	**hGALK1-galactose-T2-5**	**hGALK1-galactose-T2-6**
**Data collection**				
Beamline	DLS I04-1	DLS I04-1	DLS I04-1	DLS I04-1
Wavelength (Å)	0.91587	0.91587	0.91587	0.91587
Space group	*P* 1 21 1	*P* 1 21 1	*P* 1 21 1	*P* 1 21 1
Unit cell parameters (Å, °)	a=73.6, b=114.5, c=121.0; α =γ=90.0, β=100.4	a=73.7, b=114.6, c=120.9; α =γ=90.0, β=100.6	a=73.8, b=114.5, c=120.9; α=γ=90.0, β=100.6	a=73.7, b=114.9, c=121.0; α=γ=90.0, β=100.6
Resolution (Å)	72.4 - 2.3	118.9 - 2.3	82.6 – 1.9	67.41 - 2.4
Observed/ Unique reflections	297390/86745	299490/87629	299485/87557	261047/76815
Multiplicity	3.4 (3.6)[a]	3.4 (3.5)[a]	3.4 (3.5)[a]	3.4 (3.5)[a]
Completeness	99.1 (98.9)[a]	99.8 (99.6)[a]	99.4 (98.9)[a]	99.64 (99.79)[a]
I/sig(I)	10.3 (1.9)[a]	7.2 (1.6)[a]	11.16 (1.6)[a]	15.30 (3.64)[a]
R-merge (%)	9.0 (53.9)[a]	10.0 (62.2)[a]	8.0 (18.7)[a]	5.2 (36.2)[a]
CC (1/2) (%)	99.7 (83.4)[a]	99.4 (81.4)[a]	99.0 (76.6)[a]	99.9 (63.0)[a]
**Refinement statistics**				
Rwork (%)	20.7	20.9	24.9	22.0
Rfree (%)	24.6	25.5	27.4	27.7
R.m.s.d. bond length (Å)	0.018	0.019	0.017	0.015
R.m.s.d. bond angle (°)	2.25	2.06	2.21	1.94
PDB code	6ZGV	6ZGW	6ZGX	6Q90

[a] Values in brackets correspond to the highest resolution shell

Table S4: Crystallographic refinement statistics for hGALK1 co-crystal structures with fragments 7 – 10, binding in the allosteric hotspot.

	**hGALK1-galactose-T2-7**	**hGALK1-galactose-T2-8**	**hGALK1-galactose-T2-9**	**hGALK1-galactose-T2-10**
**Data collection**				
Beamline	DLS I04-1	DLS I04-1	DLS I04-1	DLS I04-1
Wavelength (Å)	0.91587	0.91587	0.91587	0.91587
Space group	*P* 1 21 1	*P* 1 21 1	*P* 1 21 1	*P* 1 21 1
Unit cell parameters (Å, °)	a=73.2, b=114.3, c=120.8; α =γ=90.0, β=100.5	a=73.3, b=114.6, c=120.8; α =γ=90.0, β=100.6	a=73.5, b=115.0, c=120.8; α=γ=90.0, β=100.7	a=73.3, b=114.9, c=120.9; α=γ=90.0, β=100.8
Resolution (Å)	118.8 – 2.3	118.7 - 2.3	118.8 - 2.5	82.6 - 2.4
Observed/ Unique reflections	510715/147891	296178/86820	316652/90900	263015/76670
Multiplicity	3.5 (3.4)[a]	3.4 (3.5)[a]	3.5 (3.6)[a]	3.4 (3.5)[a]
Completeness	99.6 (99.5)[a]	99.6 (99.4)[a]	99.7 (99.5)[a]	99.7 (99.1)[a]
I/sig(I)	5.8 (1.6)[a]	7.6 (1.1)[a]	4.7 (1.2)[a]	5.4 (1.15)[a]
R-merge (%)	10.8 (24.7)[a]	11.0 (39.4)[a]	13.0 (29.4)[a]	9.3 (47.1)[a]
CC (1/2) (%)	99.6 (83.5)[a]	99.1 (97.3)[a]	99.6 (74.1)[a]	99.7 (46.3)[a]
**Refinement statistics**				
Rwork (%)	21.4	20.6	23.2	24.9
Rfree (%)	25.6	25.4	25.5	27.5
R.m.s.d. bond length (Å)	0.016	0.016	0.016	0.015
R.m.s.d. bond angle (°)	2.07	2.14	2.29	2.05
PDB code	6ZGY	6ZGZ	6ZH0	6Q91

[a] Values in brackets correspond to the highest resolution shell

Table S5: Kinetic parameters calculated for inhibitors of hGALK1 from data collected in Kinase-Glo and Amplex Red assays. The apparent Vmax and *K*m values are shown for hGALK1 in saturating concentrations of galactose (top) and ATP (bottom) without compound and at the maximum tested compound concentration. The ATP concentration at which galactose kinetics were measured, using the Kinase-Glo assay, was 35 µM. The galactose concentration at which ATP kinetics were measured, using the Amplex Red assay, was 50 µM. The maximum tested concentrations were 1 mM for compound **T1**, 15 mM for fragment **3** and 2 mM for compounds **11** – **13**.

**Substrate**: **Galactose**				
Parameter:	$Vmax_{app}$		Km_{app}	
Ligand:	-	+	-	+
**T1**	14.4 ± 2.1	4.1 ± 0.5	33.9 ± 3.2	32.9 ± 1.0
Fragment **3**	27.8 ± 1.6	5.1 ± 0.4	30.6 ± 1.3	184.2 ± 2.1
**11**	19.5 ± 34.9	3.7 ± 0.4	33.9 ± 0.8	36.5 ± 2.1
**12**	28.5 ± 1.6	9.4 ± 1.1	34.5 ± 5.0	41.5 ± 12.4
**13**	16.9 ± 0.7	4.4 ± 0.4	34.9 ± 9.5	143.6 ± 7.6
**Substrate**: **ATP**				
Parameter:	$Vmax_{app}$		Km_{app}	
Ligand:	-	+	-	+
**T1**	27.9 ± 2.1	27.9 ± 1.5	39.9 ± 0.5	63.5 ± 2.6
Fragment **3**	15.7 ± 2.2	4.2 ± 0.3	40.5 ± 3.8	32.1 ± 8.4
**11**	30.2 ± 3.3	11.1 ± 0.9	40.9 ± 4.5	36.5 ± 5.8
**12**	28.7 ± 0.6	11.8 ± 0.2	33.4 ± 0.5	40.4 ± 4.7
**13**	32.0 ± 1.8	14.7 ± 3.0	41.6 ± 3.6	46.5 ± 4.5

Table S6: IC_{50} and LLE_{AT} scores for 11 and 12, as calculated in Kinase-Glo and Amplex Red assays. Results are for two biological replicates, labelled N1 and N2 respectively, and the LLE_{AT} is calculated cumulatively. IC_{50} values are given in μM and the units for LLE_{AT} scores are kcal/mol.

	**Kinase-Glo**			**Amplex Red**		
	**N1 IC_{50}**	**N2 IC_{50}**	**LLE_{AT}**	**N1 IC_{50}**	**N2 IC_{50}**	**LLE_{AT}**
**11**	209	116	0.19	31	65	0.22
**12**	198	96	0.23	45	25	0.27

Table S7: ICM 4D docking results for compound 11. Scoring of ICM docking parameters for the 10 binding conformations of compound **11** that had an overall score better than -20.

	**ICM Score**	**Ligand strain (kcal/mol)**	**Van der Waals**	**Solvation electrostatics**	**H-Bond energy**	**Hydrophobic energy**	**Desolvation of exposed HBD/HBA**
**1***	-27.01	4.06	-30.14	7.79	-5.77	-7.01	15.23
**2**	-26.21	2.80	-32.55	10.40	-4.66	-6.79	14.96
**3**	-26.09	2.20	-26.73	5.55	-5.16	-6.79	14.10
**4**	-25.83	3.68	-29.42	6.00	-4.79	-6.71	14.36
**5**	-23.86	3.38	-32.40	9.51	-3.55	-7.05	14.41
**6**	-23.48	2.89	-31.35	9.68	-2.32	-7.03	12.83
**7**	-22.34	5.07	-26.53	5.78	-4.52	-7.07	12.93
**8**	-22.01	5.50	-30.56	9.18	-3.87	-7.08	12.96
**9**	-21.97	3.40	-28.66	7.35	-3.07	-6.73	11.88
**10**	-21.38	4.10	-28.04	7.08	-3.22	-6.81	11.72

*referred to in text as the top scored binding conformation

Table S8: ICM 4D docking results for compound 12. Scoring of ICM docking parameters for the 14 binding conformations of compound **12** that had an overall score better than -20.

	ICM Score	Ligand strain (kcal/mol)	Van der Waals	Solvation electrostatics	H-Bond energy	Hydrophobic energy	Desolvation of exposed HBD/HBA
**1***	-33.06	2.57	-40.17	6.00	-2.52	-7.96	12.64
**2**	-30.72	4.08	-33.58	6.93	-6.52	-7.02	16.55
**3**	-30.53	7.08	-30.78	5.95	-8.41	-7.16	16.43
**4**	-30.40	5.09	-32.35	7.70	-7.40	-7.06	16.30
**5**	-25.93	3.17	-33.66	6.96	-4.17	-6.96	16.24
**6**	-25.90	5.64	-32.88	8.22	-5.62	-7.13	15.88
**7**	-23.69	3.45	-32.58	8.76	-4.37	-7.09	16.61
**8**	-23.25	7.35	-32.29	8.42	-5.58	-7.04	15.93
**9**	-22.61	3.67	-31.16	8.80	-4.90	-6.90	17.44
**10**	-22.53	2.60	-34.28	8.33	-2.61	-6.99	15.92
**11**	-22.19	3.28	-26.77	7.89	-5.48	-7.23	15.73
**12**	-21.07	3.77	-31.32	10.51	-4.31	-7.10	16.26
**13**	-20.42	2.88	-32.14	7.90	-2.30	-7.20	15.01
**14**	-20.02	4.71	-33.14	7.97	-2.78	-6.96	15.78

*referred to in text as the top scored binding conformation

Table S9: ICM 4D docking results for compound 13. Scoring of ICM docking parameters for the 8 binding conformations of compound **13** that had an overall score better than -20.

	ICM Score	Ligand strain (kcal/mol)	Van der Waals	Solvation electrostatics	H-Bond energy	Hydrophobic energy	Desolvation of exposed HBD/HBA
**1***	-32.28	4.31	-32.75	8.71	-7.13	-6.96	15.85
**2**	-31.55	9.78	-39.45	9.28	-5.16	-7.85	12.96
**3**	-31.11	4.79	-39.62	8.41	-2.80	-7.85	13.20
**4**	-26.28	4.73	-36.26	10.82	-2.92	-7.91	13.66
**5**	-25.01	6.86	-33.73	9.65	-4.12	-7.82	14.08
**6**	-25.01	3.81	-36.47	11.44	-2.37	-7.93	14.42
**7**	-22.12	7.04	-35.23	11.65	-3.12	-7.82	14.56
**8**	-21.49	5.58	-34.21	10.35	-2.43	-7.94	14.91

*referred to in text as the top scored binding conformation

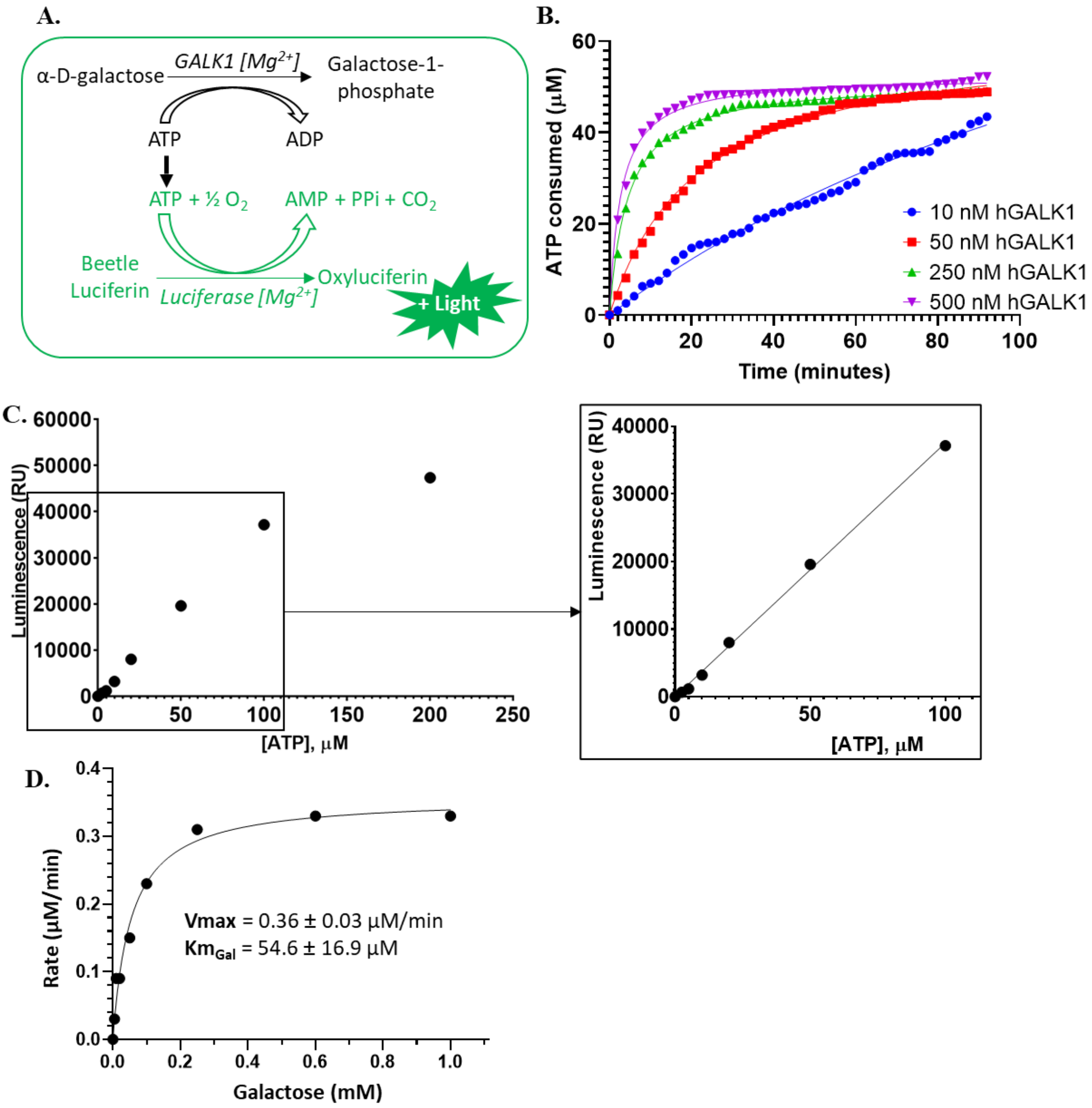

Figure S1: Assay set up and validation for Kinase-Glo hGALK1 activity assay. (A) Schematic showing how hGALK1 activity is measured using the Kinase-Glo assay. The hGALK1 reaction is shown in black text and the Kinase-Glo reaction is in green text. (B) Plot showing ATP consumed by 10 nM (blue), 50 nM (red), 250 nM (green) or 500 nM (purple) hGALK1 over time. Reaction conditions were 50 µM ATP and 100 µM galactose. (C) Plot showing luminescence signal against increasing ATP concentration, up to 200 µM ATP. *Inset:* Close-up view of the linear range for luminescence signal against ATP concentration, up to 100 µM ATP. (D) Michaelis-Menten plot showing the reaction rate of hGALK1 (µM ATP consumed per minute) in the presence of increasing galactose concentrations up to 1 mM. Reaction conditions were 10 nM hGALK1 and 35 µM ATP and reaction rate was calculated for the 1 hour incubation of hGALK1 and substrate prior to addition of the Kinase-Glo reagent.

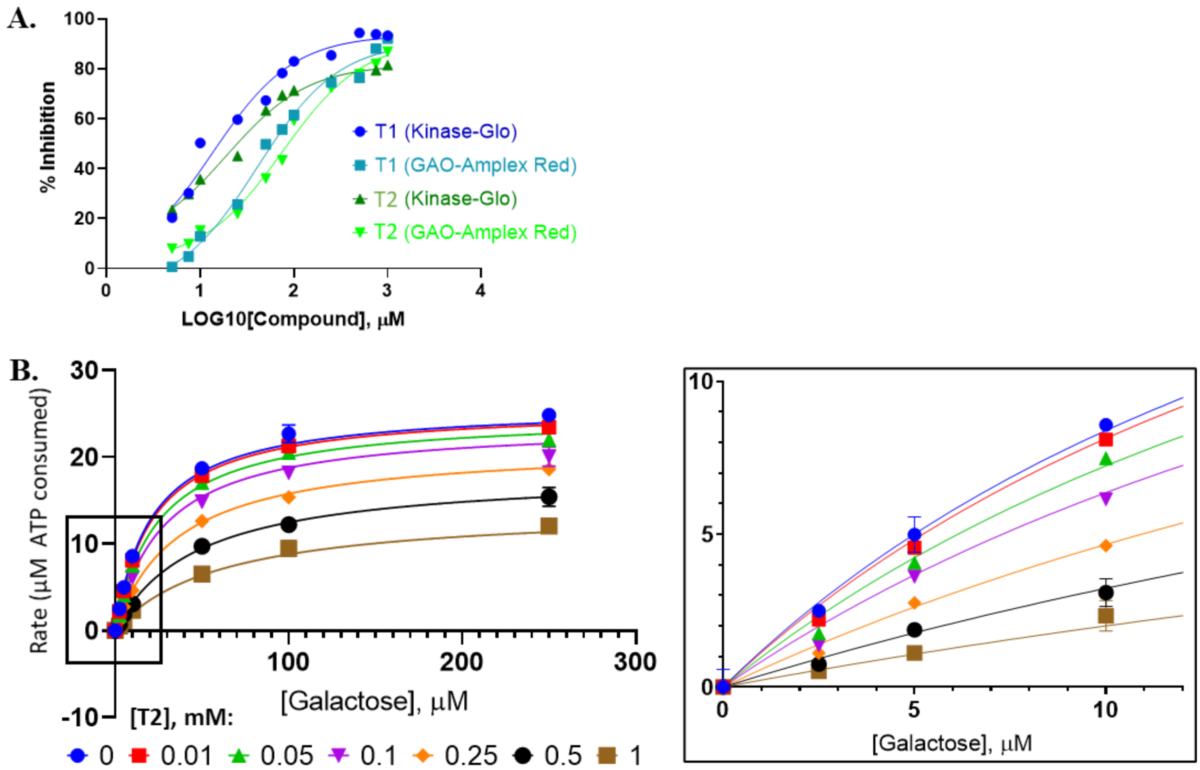

Figure S2: Characterisation of hGALK1 inhibition by spiro-benzoxazole inhibitors T1 and T2. (A) Concentration-response curves for spiro-benzoxazole compounds **T1** (blue) and **T2** (green). Representative curves from the Kinase-Glo assay are in dark shades and curves from the Amplex Red assay are in light shades. The reaction conditions in the former are 10 nM hGALK1, 35 μM ATP and 100 μM galactose and the reaction conditions in the latter are 250 nM hGALK1, 100 μM ATP and 50 μM galactose. (B) Least-squares non-linear fit of GALK1 reaction rate (total ATP consumed after 1 hour reaction, μM) against increasing galactose concentrations (0 – 250 μM) in the presence of different concentrations of the inhibitor **T2** (0 – 1 mM). Curves were fitted to non-competitive inhibition model, the best fitting Enzyme kinetics – Inhibition equations for the data, using the GraphPad Prism software. *Inset*: Close-up view of plot showing GALK1 reaction rate (total ATP consumed after 1 hour reaction, μM) against increasing galactose concentrations (0 – 10 μM) in the presence of different concentrations of the inhibitor **T2** (0 – 1 mM).

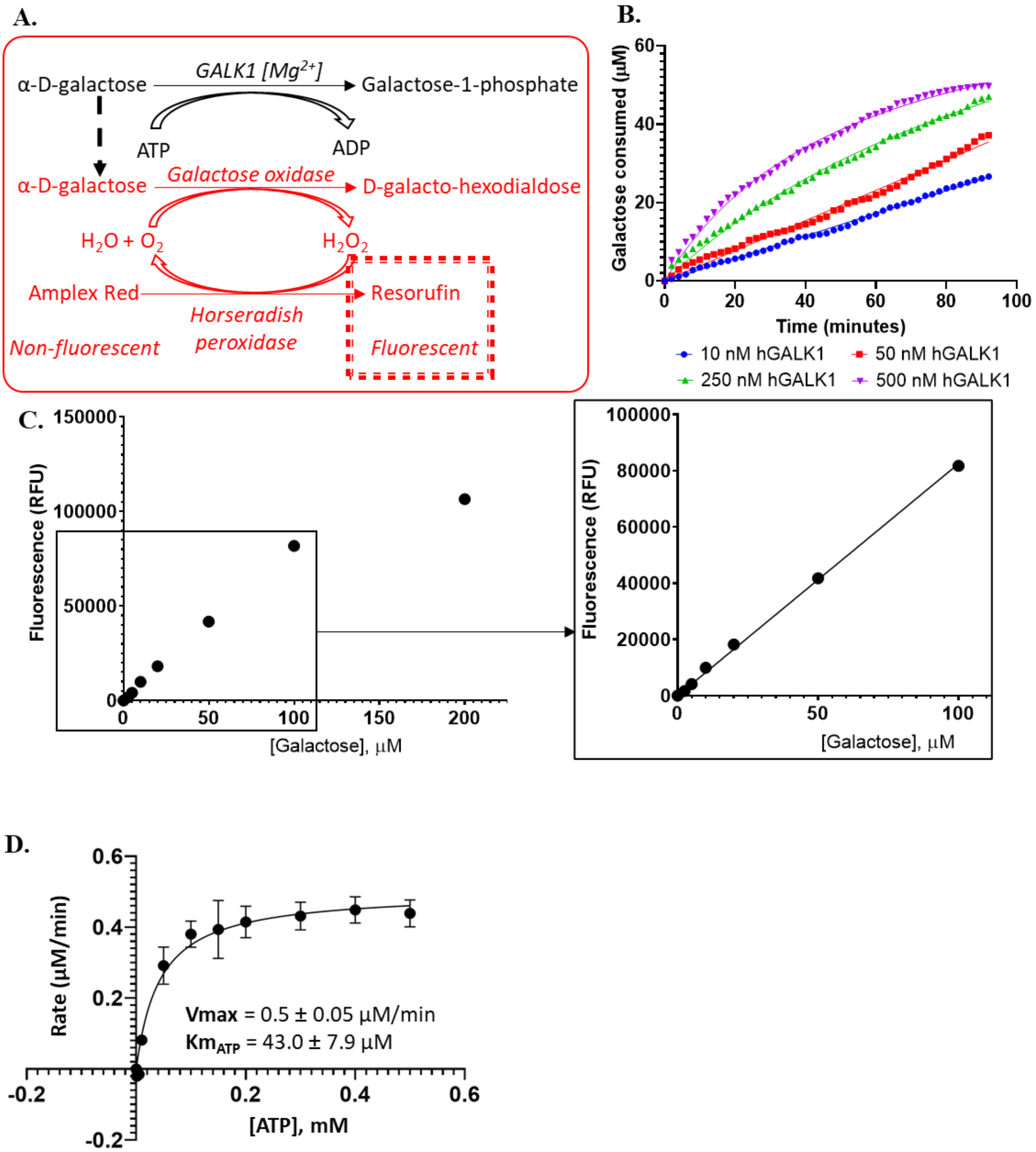

Figure S3: Assay set up and validation for Amplex Red hGALK1 activity assay. (A) Schematic showing how hGALK1 activity is measured using the Amplex Red assay. The hGALK1 reaction is shown in black text and the Amplex Red reaction is in red text. (B) Plot showing galactose consumed by 10 nM (blue), 50 nM (red), 250 nM (green) or 500 nM (purple) hGALK1 over time. Reaction conditions were 100 µM ATP and 50 µM galactose. (C) Plot showing fluorescence signal against increasing galactose concentration, up to 200 µM galactose. *Inset:* Close-up view of the linear range for fluorescence signal against galactose concentration, up to 100 µM galactose. (D) Michaelis-Menten plot showing the reaction rate of hGALK1 (µM galactose consumed per minute) in the presence of increasing ATP concentrations up to 0.5 mM. Reaction conditions were 250 nM hGALK1 and 50 µM galactose and reaction rate was calculated for the 60 minutes incubation of hGALK1 and substrate prior to addition of the Amplex Red reagent.

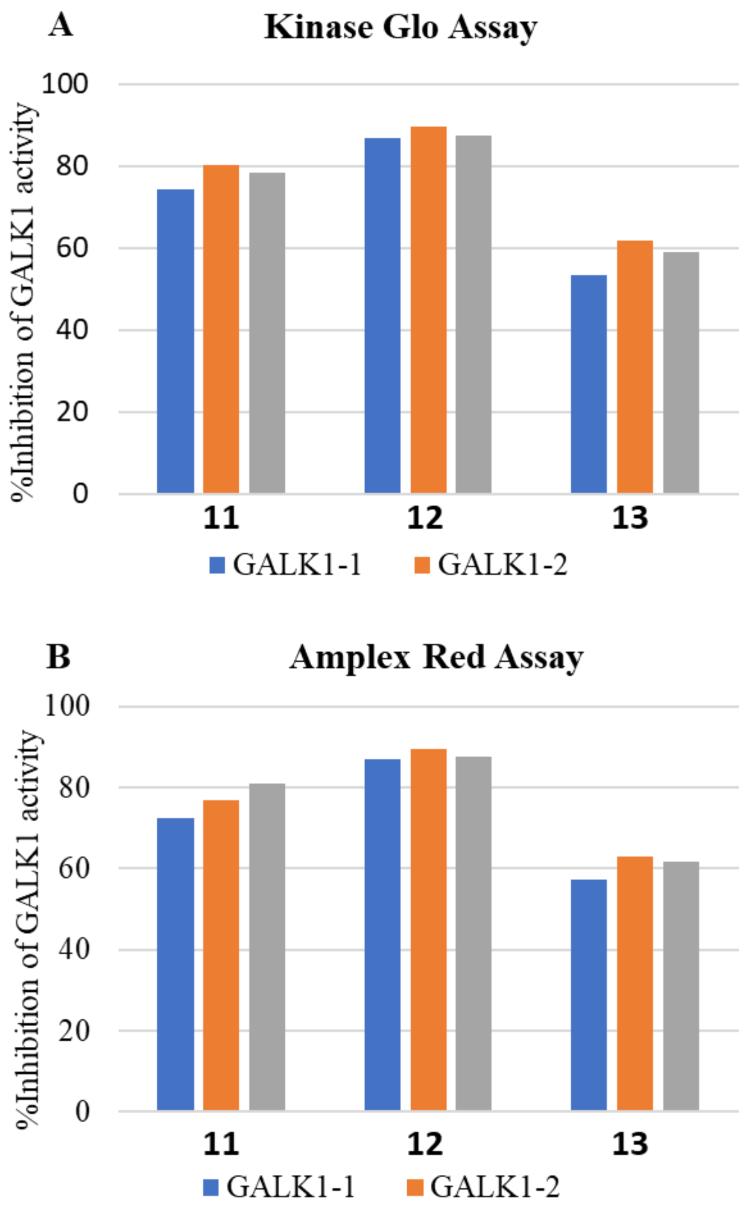

Figure S4: Inhibition of hGALK1 by 2 mM of follow-up compounds 11 – 13. (A) Bar chart showing inhibition of hGALK1 by 2 mM follow-up compound **11** – **13**, measured in the Kinase-Glo assay as described in the text. (B) Bar chart showing inhibition of hGALK1 by 2 mM follow-up compound **11** – **13**, measured in the Amplex Red assay as described in the text.

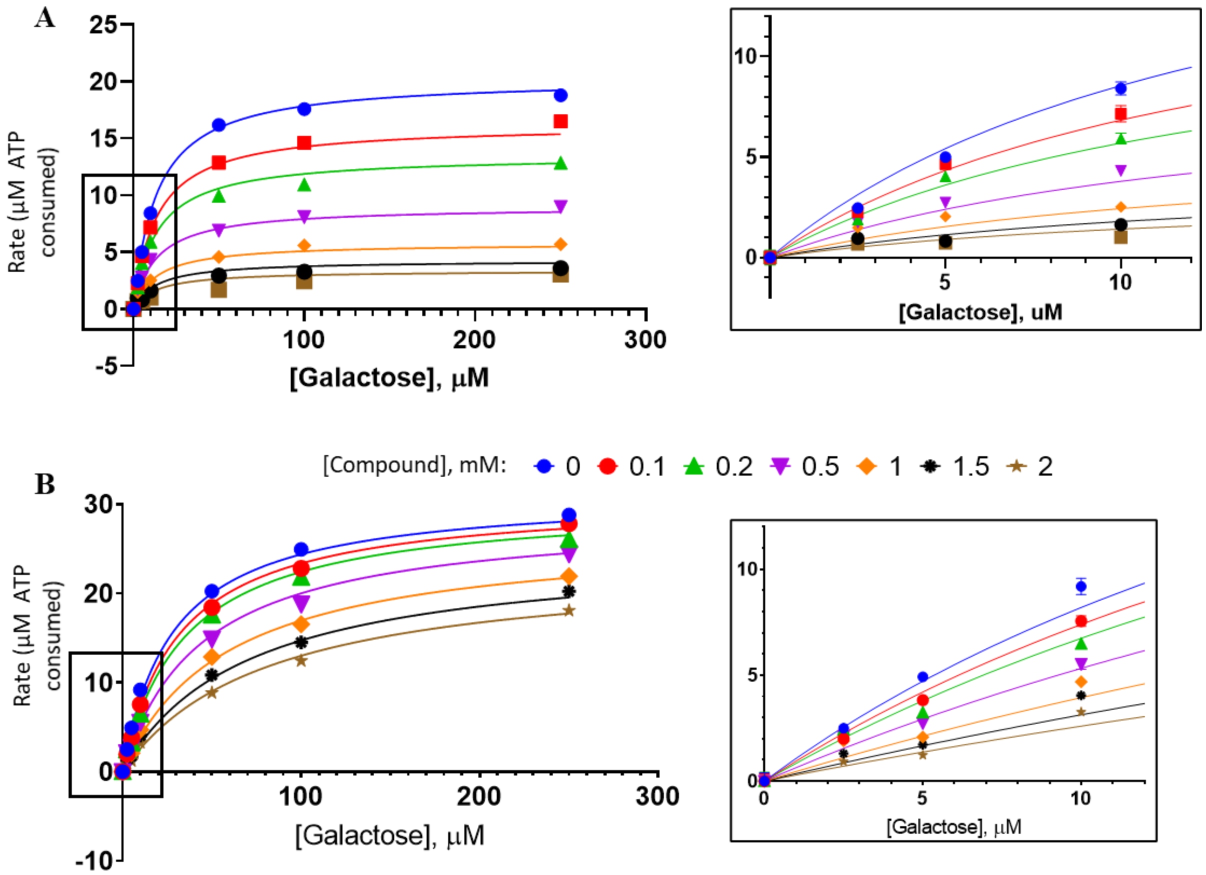

Figure S5: Inhibition mode of follow-up compounds 11 and 13 with respect to galactose. Least-squares non-linear fit of GALK1 reaction rate (total ATP consumed after 1 hour reaction, µM) against increasing galactose concentrations (0 – 250 µM) in the presence of different concentrations (0 – 2 mM) of **11** (A) and **13** (B), as determined in the Kinase-Glo assay. Compound **11** curves were fitted to a non-competitive inhibition model while compound **13** curves were fitted to a mixed inhibition model, the best fitting Enzyme kinetics – Inhibition equations for the data, using the GraphPad Prism software. *Inset*: Close-up view of plot showing GALK1 reaction rate (total ATP consumed after 1 hour reaction, µM) against increasing galactose concentrations (0 – 10 µM) in the presence of different concentrations (0 – 2 mM) of **11** (A) and **13** (B).

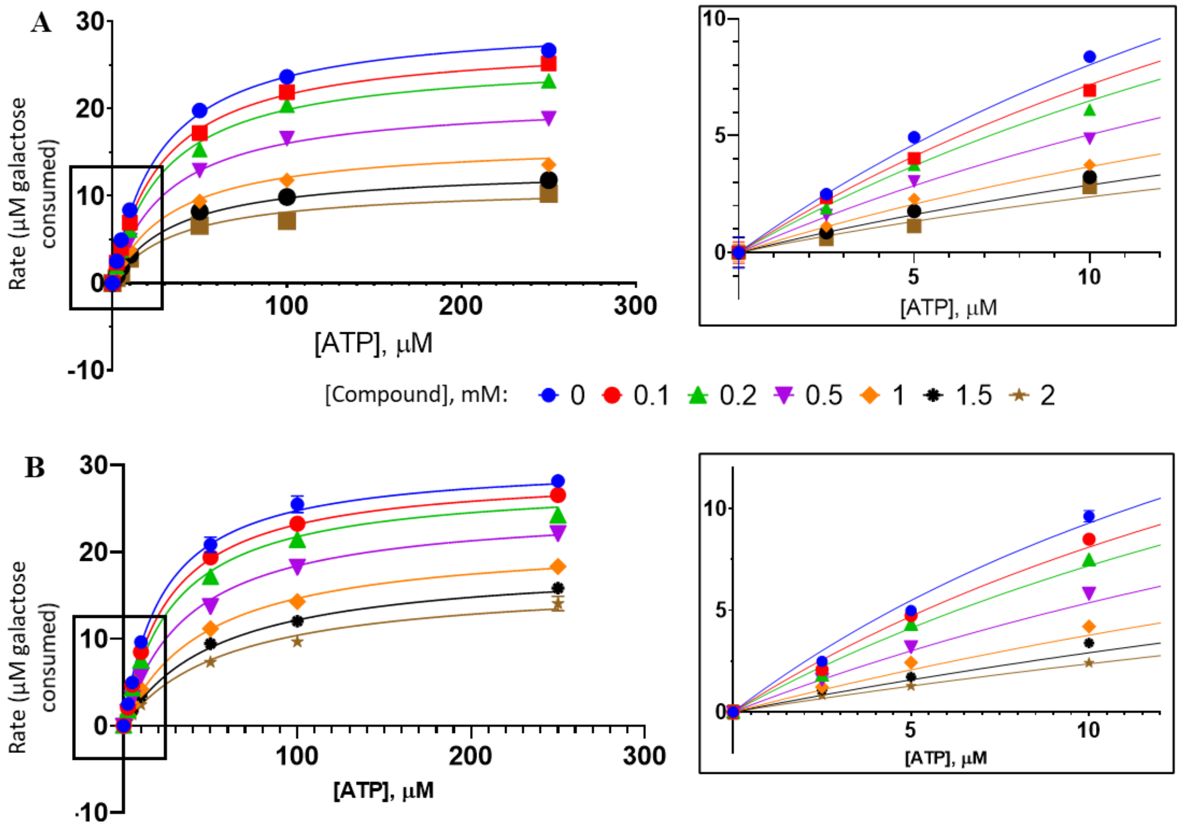

Figure S6: Inhibition mode of follow-up compounds 11 and 13 with respect to ATP. Least-squares non-linear fit of GALK1 reaction rate (total galactose consumed after 1 hour reaction, μM) against increasing ATP concentrations (0 – 250 μM) in the presence of different concentrations (0 – 2 mM) of **11** (A) and **13** (B), as determined in the Amplex Red assay. Curves were fitted to non-competitive inhibition model, the best fitting Enzyme kinetics – Inhibition equation, using the GraphPad Prism software. Close-up view of plot showing GALK1 reaction rate (total galactose consumed after 1 hour reaction, μM) against increasing ATP concentrations (0 – 10 μM) in the presence of different concentrations (0 – 2 mM) of **11** (A) and **13** (B). .

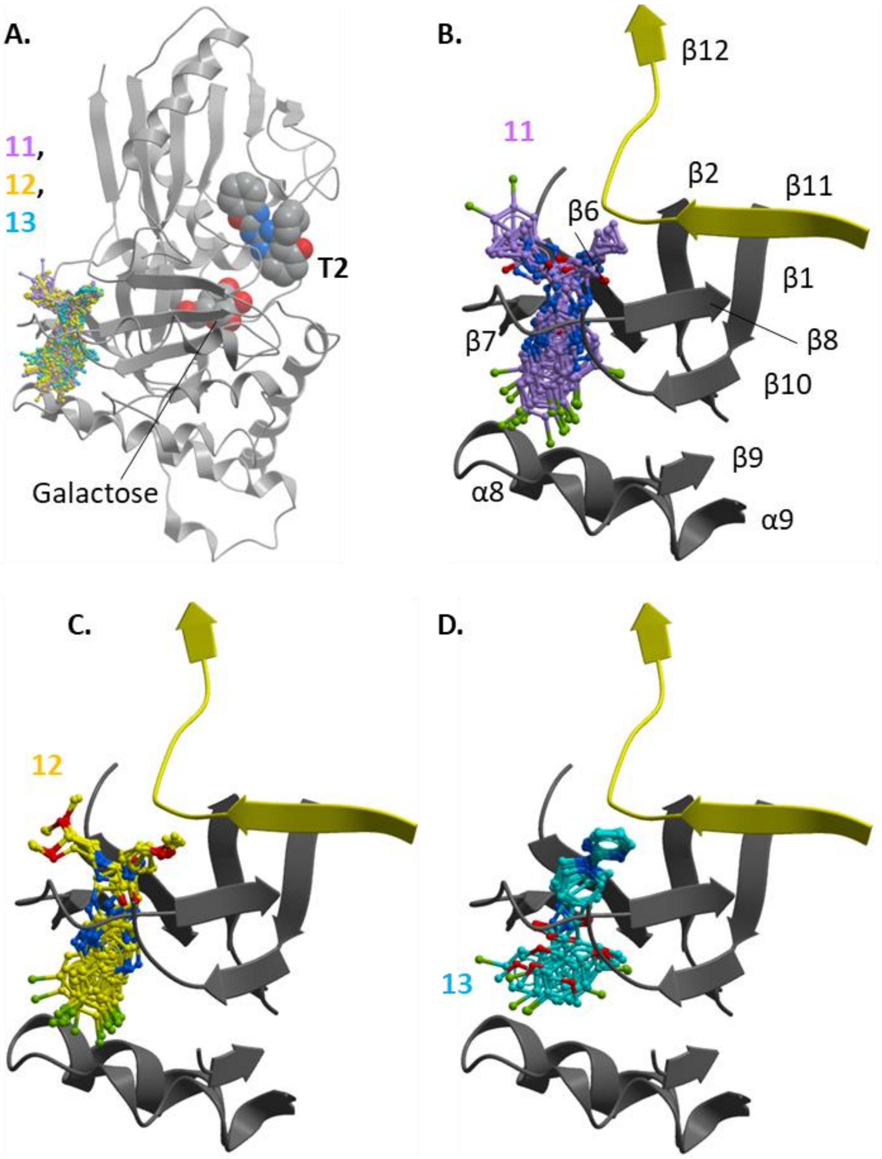

Figure S7: Docking of compounds 11-13 to hGALK1 flexible receptor. A. Ribbon diagram of hGALK1-galactose-**T2**-fragment **10** structure, the hGALK1 structure yielding the most favourable docking scores, showing the location of 23, 33 and 11 docked conformations of compounds **11, 12** and **13** respectively. Galactose and **T2** are shown as spacefill and compounds **11-13** are shown as lilac, yellow and blue sticks, respectively. (B-D). Close-up view of the binding hotspot of hGALK1-galactose-**T2**-fragment **10** structure with compound **11** (B), compound **12** (C) and compound **13** (D) docked conformations that had an ICM score better than -20 (10, 14 and 8 conformations respectively). Secondary structure elements of the binding hotspot (as defined in the Results and Discussion section) are shown as grey ribbon and the additional interacting β11-β12 loop is shown as yellow ribbon.

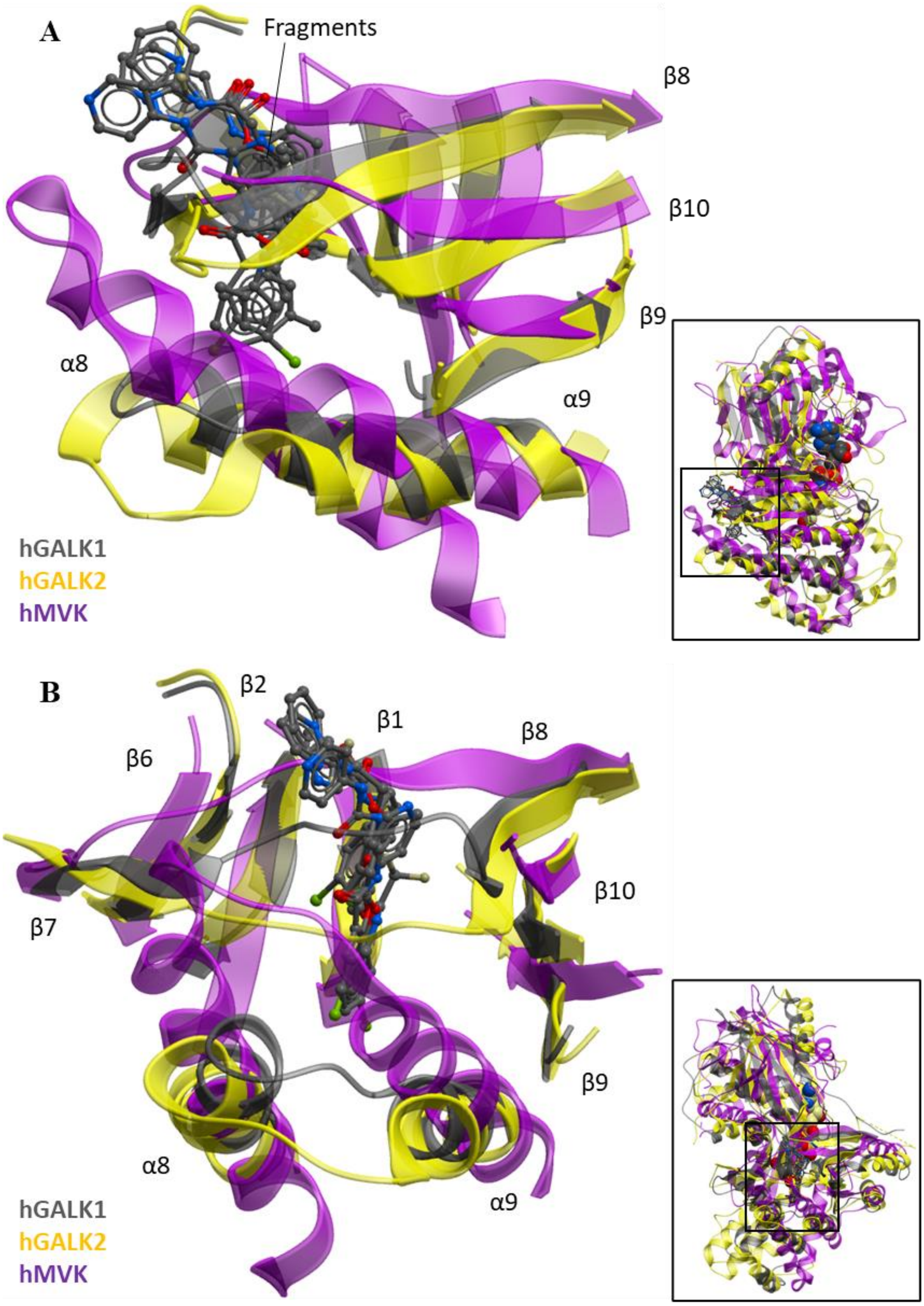

Figure S8: Structural alignment of identified hotspot in hGALK1, with equivalent structural elements in hGALK2 and hMVK. Close-up side (A) and front (B) views of superimposed structures of hGALK1-galactose-**T2** (solved in this work, grey), hGALK2-N-acetylgalactosamine-Mn-AMPPNP (PDB code 2a2d, yellow) and hMVK (PDB code 2r3v, purple) at the binding hotspot identified in this work, displayed as ribbons. Fragments **3** – **10** are shown as sticks and key secondary structure elements (GALK1 naming) are labelled. *Inset:* Overall structure of superimposed GHMP kinases illustrating the viewpoint of the adjacent close-up view.

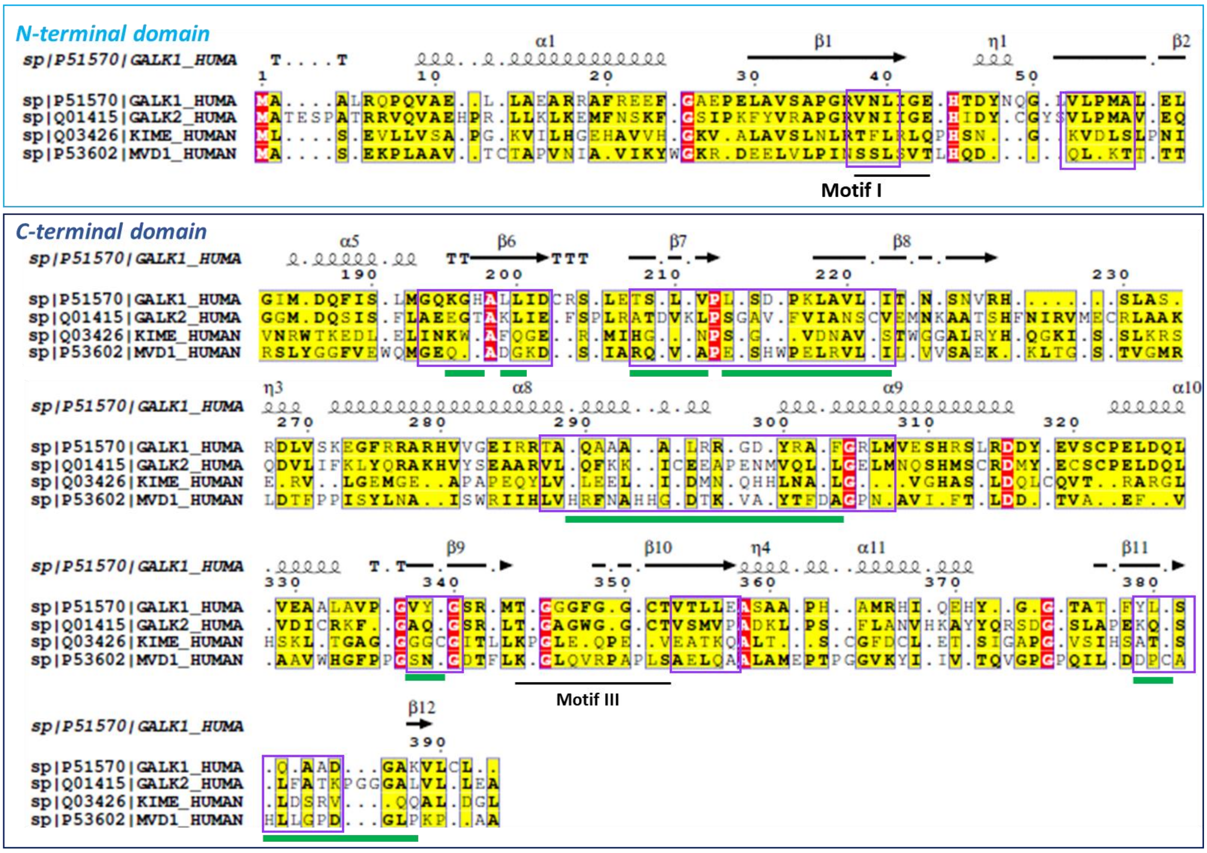

Figure S9: Sequence alignment of GHMP kinase family members comparing secondary structures at the binding hotspot of hGALK1. Invariant residues are shown as white characters on a red background, residues that are similar in >70% of sequences are shown as bold characters on a yellow background and residues that are similar in ≥50% of sequences also have a yellow background. The GHMP kinase motifs are underlined in black and secondary structure features of the hotspot site are shown in purple boxes. Variable regions are underlined in green.

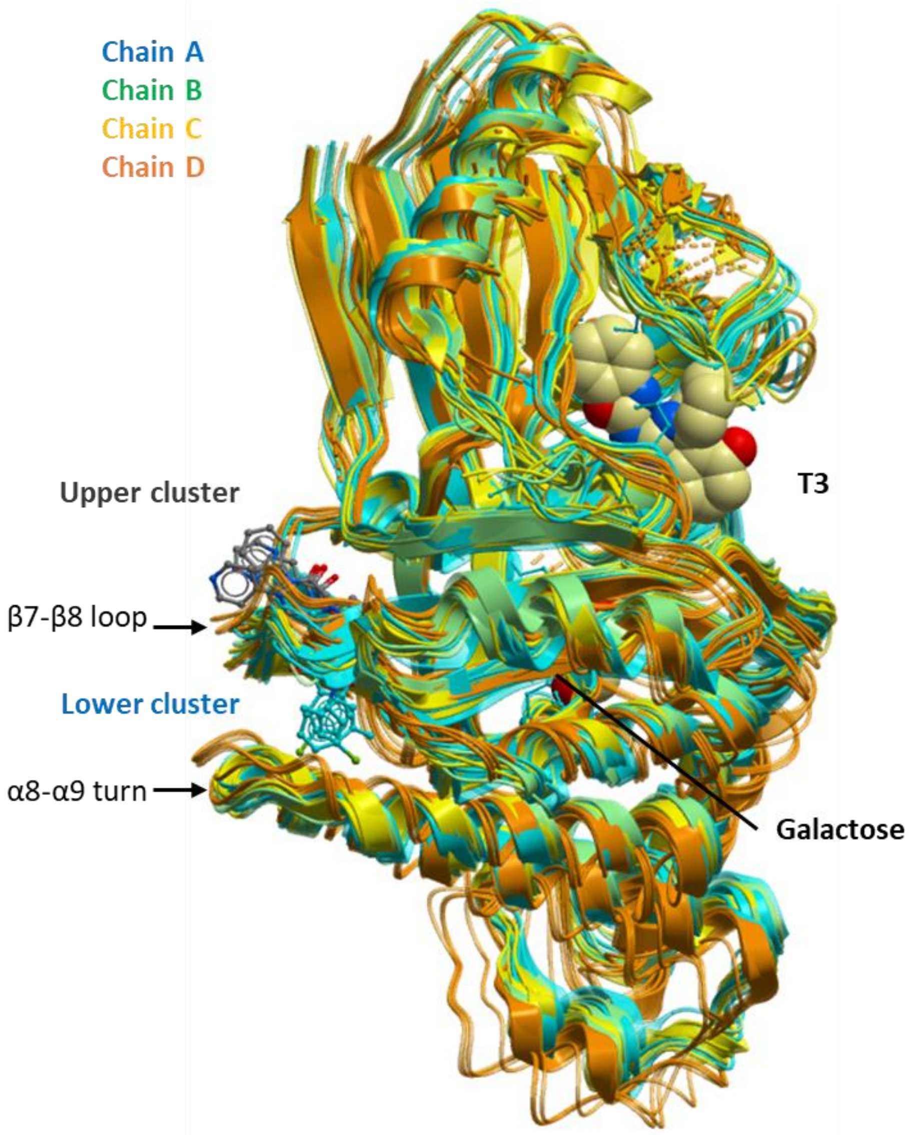

Figure S10: Superimposition of the four protein chains from GALK1 fragment-bound structures. Ribbon diagram showing superimposed structures of all protein chains A (blue), B (green), C (yellow) and D (orange) from GALK1 co-crystal structures with binding hotspot fragments **3** – **10**. Galactose and compound **T2** are shown as spacefill. Fragments belonging to the upper cluster (**3** – **6**) are shown as grey sticks and fragments belonging to the lower cluster (**7** – **10**) are shown as blue sticks). The two most variable regions, the β7-β8 loop and the α8-α9 turn are indicated with arrows.

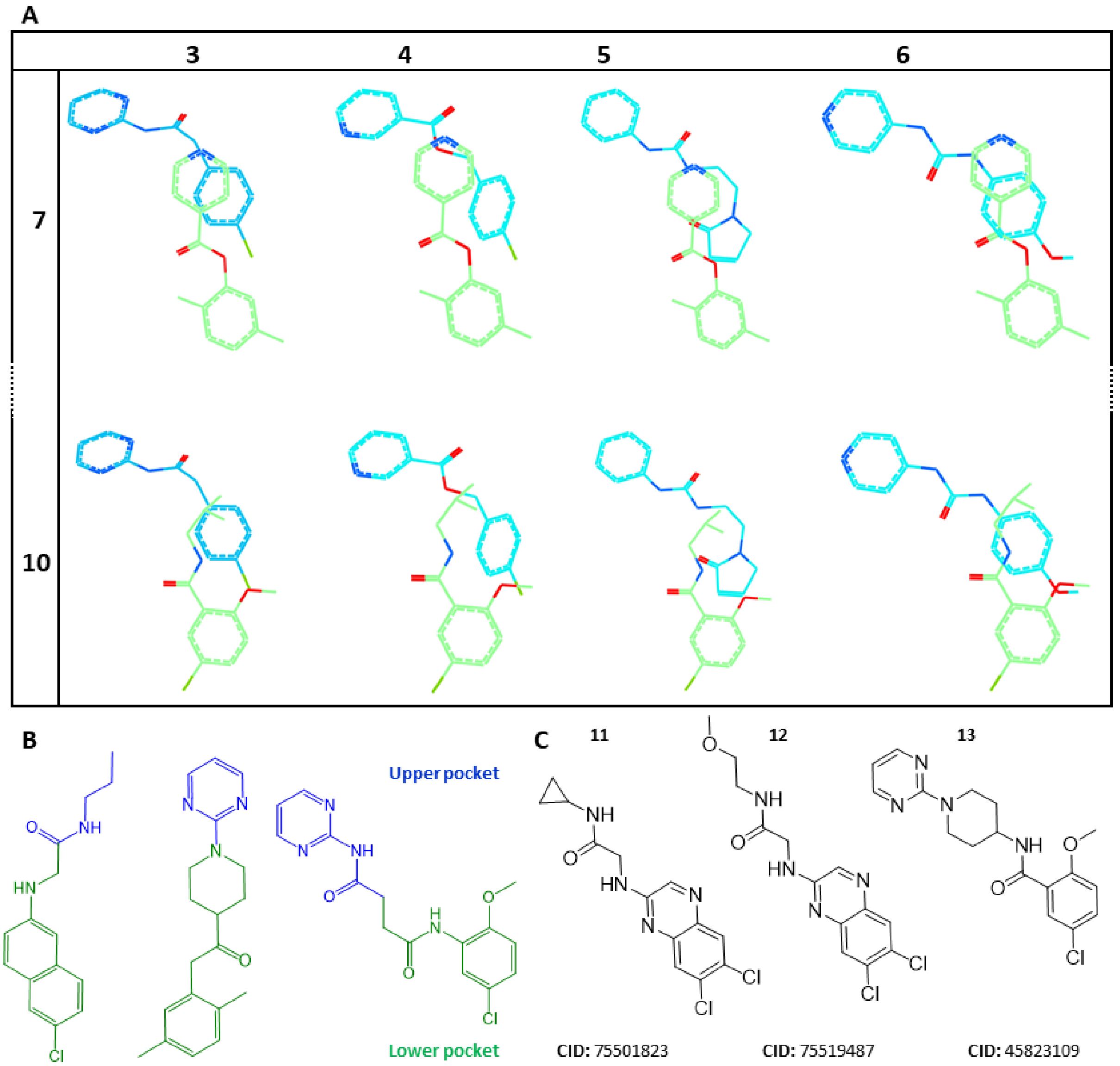

Figure S11: Experimental, theoretical, and purchased follow-up compounds derived from allosteric hotspot fragments. (A) Representative overlaid fragment chemical structures as observed upon superimposition of fragment-bound hGALK1 structures. Upper cluster fragments (**3** – **6**) are shown as blue lines and lower cluster fragments (**7**, **10**) are shown as green lines. (B) Theoretical compounds drawn to recapitulate overlapping chemical groups from fragments. (C) Commercially available compounds **11** -**13**, tested in this work.
